# Discovery and partial characterization of a non-LTR retrotransposon that may be associated with abdominal segment deformity disease (ASDD) in the whiteleg shrimp *Penaeus (Litopenaeus) vannamei*

**DOI:** 10.1186/1746-6148-9-189

**Published:** 2013-09-30

**Authors:** Waraporn Sakaew, Benjamart Pratoomthai, Pattira Pongtippatee, Timothy W Flegel, Boonsirm Withyachumnarnkul

**Affiliations:** 1 Department of Anatomy, Faculty of Science, Mahidol University, Bangkok 10400, Thailand; 2 Center of Excellence for Shrimp Molecular Biology and Biotechnology (Centex Shrimp), Faculty of Science, Mahidol University, Bangkok 10400, Thailand; 3 Aquatic Animal Biotechnology Research Center, Faculty of Science and Industrial Technology, Prince of Songkla University Surat Thani, Surat Thani 84100, Thailand; 4 Department of Biotechnology, Faculty of Science, Mahidol University, Bangkok 10400, Thailand; 5 National Center for Genetic Engineering and Biotechnology (BIOTEC), National Science and Technology Development Agency, Klong 1Klong Luang, Pratum Thani 12120, Thailand

## Abstract

**Background:**

Abdominal segment deformity disease (ASDD) of cultivated whiteleg shrimp *Penaeus (Litopenaeus) vannamei* causes economic loss of approximately 10% in affected specimens because of the unsightliness of distorted abdominal muscles. It is associated with the presence of viral-like particles seen by electron microscopy in the ventral nerve cords of affected shrimp. Thus, shotgun cloning was carried out to seek viral-like sequences in affected shrimp.

**Results:**

A new retrovirus-like element of 5052 bp (named abdominal segment deformity element or ASDE) was compiled by shotgun cloning and 3′ and 5′ RACE using RNA and DNA extracted from ventral nerve cords of ASDD shrimp. ASDE contained 7 putative open reading frames (ORF). One ORF (called the PENS sub-domain), had a deduced amino acid (aa) sequence homologous to the GIY-YIG endonuclease domain of penelope-like retrotransposons while two others were homologous to the reverse transcriptase (RT) and RNaseH domains of the pol gene of non-long terminal repeat (non-LTR) retrotransposons (called the NLRS sub-domain). No single amplicon of 5 kb containing both these elements was obtained by PCR or RT-PCR from ASDD shrimp. Subsequent analysis indicated that PENS and NLRS were not contiguous and that NLRS was a host genetic element. *In situ* hybridization using a dioxygenin-labeled NLRS probe revealed that NLRS gave positive reactions in abdominal-ganglion neurons of ASDD shrimp *but not* normal shrimp. Preliminary analysis indicated that long-term use of female broodstock after eyestalk ablation in the hatchery increased the intensity of RT-PCR amplicons for NLRS and also the prevalence of ASDD in mysis 3 offspring of the broodstock. The deformities persist upon further cultivation until shrimp harvest but do not increase in prevalence and do not affect growth or survival.

**Conclusions:**

Our results suggested that NLRS is a shrimp genetic element associated with ASDD and that immediate preventative measures could include shorter-term use of broodstock after eyestalk ablation and/or discard of broodstock that give strong RT-PCR reactions for NLRS. In the longer term, it is recommended, if possible, that currently used, domesticated shrimp lines be selected for freedom from NLRS. The molecular tools developed in this work will facilitate the management and further study of ASDD.

## Background

Abdominal segment deformity disease (ASDD) has been reported in cultured whiteleg shrimp *Penaeus* (*Litopenaeus*) *vannamei* in Thailand, Malaysia and Indonesia [1]. The affected shrimp have deformed abdominal segments accompanied by muscle necrosis and degeneration, and also show the presence of non-enveloped viral-like particles (20–22 nm) in muscles, gills and ventral nerve cords. Shrimp viruses such as infectious hypodermal and haematopoietic necrosis virus (IHHNV) (also now known as *Penaeus stylirostris* densovirus or *Pst*DNV) and infectious myonecrosis virus (IMNV) previously known to cause physical deformity and muscle abnormality in shrimp were ruled out as causative agents of ASDD by negative findings using specific polymerase chain reaction (PCR) and *in situ* hybridization methods.

Since ASDD has no effect on shrimp growth rate or survival, it has been considered a relatively minor problem in terms of economic loss in comparison to lethal shrimp viruses such as white spot syndrome virus (WSSV). However, the value of distorted shrimp is approximately 10% less than normal shrimp and farmer losses are proportional to the fraction of a crop that is affected [1]. The impact can be significant, especially when market fluctuations lead to low profit margins. Thus, further investigation into the cause and possible prevention measures was justified to improve production efficiency. Because the initial investigation of ASDD revealed the presence of viral-like particles, it was of interest to determine whether any virus or viral-like agent could be identified from diseased shrimp. In this report, we describe the presence of a retroviral-like element that appeared to be causally linked to axonal degeneration leading to muscle atrophy and distorted abdominal segments in *P. vannamei*. Since the agent appeared to be transmitted from broodstock to their offspring, recommended precautionary strategy should focus on immediate management and monitoring of broodstock and, if possible, long-term selection of stocks free of the element.

## Results

### Shotgun cloning

From shotgun cloning, a DNA library of 84 clones was obtained. After screening by dot blot hybridization using shrimp DNA DIG-labeled probe, 11 clones were immediately discarded because of strong cross hybridization with a DIG-labeled DNA probe from normal shrimp. Of the remaining 73 clones, 65 gave weak hybridization signals with the same probe while 8 did not hybridize. Sequencing of these 73 clones followed by BLASTn and BLASTx searches revealed that 5 had significant homology to shrimp immune genes, 13 to mitochondrial or nuclear genes of penaeid shrimp and 23 to hypothetical proteins of various other organisms (total 42 clones). The remaining 31clones showed no similarity to known sequences and only 1 clone of 1316 bp had a deduced amino acid sequence (frame+2) with homology to a virus-like, pol protein (BgI) of a non-LTR retrotransposon of the gastropod mollusk *Biomphalaria glabrata* (GenBank: ABN58714, E value 4e-^61^ at 30% identity and 88% coverage) and to a sequence of *Drosophila melanogaster* (GenBank: CAC16871.1, Expect = 6e-44 with 26% identity and 94% coverage). Its nucleic acid sequence also shared high identity (93%) with a *P. vannamei* expressed sequence tag (EST) (MGID512728) (Additional file 1) and less identity (81%) to a *P. monodon* EST (MGID126456). No significant homology was found for other retrotransposable elements previously described from penaeid shrimp [2]. For convenience herein, this clone and its extended sequence by RACE will be referred to as abdominal segment deformity element (ASDE).

### Rapid amplification of cDNA ends (RACE)

**Figure 1:**
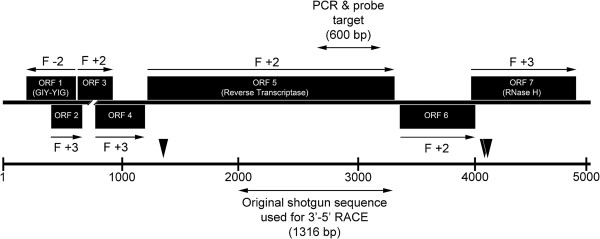
**The genomic organization of ASDE (5052 bp).** Reading frame −2 encodes for the GIY-YIG endonuclease. Reading frame +2 encodes for the reverse transcriptase (RT) portion and reading frame +3 for the RNaseH portion of the pol-like protein gene. The location of the original shotgun clone sequence used or 3′-5′ RACE is indicated next to the underlying scale bar for the whole sequence of 5052 bp. Also shown is the location of the 600 bp target sequence for the PCR, RT-PCR and hybridization assays. The black arrowheads indicate the positions of BamH1 cutting sites at positions 1373, 4137 and 4145.

By the RACE technique, the original ASDE sequence of 1,316 bp was extended to 5,052 bp. Analysis of the extended sequence by ORF finder (GenBank) indicated 7 putative ORF (Figure 1 and Additional file 2). The deduced amino acid sequences of ORFs 1, 4, 5, 6 and 7 gave significant homology to known sequences at GenBank using BLASTp. That of ORF 1 (frame −2, bases 234–632 = 132 aa) gave a hit (GenBank: EFN65003.1, Expect = 3e-11 at 35% identity and 63% coverage) for a carpenter ant (*Camponotus floridanus*) hypothetical protein that contained a conserved GIY-YIG endonuclease domain characteristic of penelope-like retrotransposons [3]. In addition, a tBLASTn search of crustacean EST records for this ORF gave a hit from *P. vannamei* hepatopancreatic cDNA (e.g., FE136984.1, Expect = 2e-63 at 89% identity and 77% coverage) and from *P. monodon* post larvae (GO072168.1, Expect = 1e-35 at 62% identity and 64% coverage). ORF 4 gave a hit with an amphipod protein of unknown function (XP003389240.1, Expect = 8e-04 at 31% identity) and with and EST sequence of *P. vannamei* (CK591146.1, Expect = 1e-25 at 72% identity and 61% coverage). ORF 5 (frame +2, bases 1250–3400 = 716 aa) gave BLASTp hits for pol regions of non-LTR retrotransposons containing conserved domains for reverse transcriptase (RT) in *B. glabrata* and *D. melanogaster*, as described above (Additional file 2), but it also gave tBLASTn hits for EST records of *P. vannamei* (CK571996.1, Expect = 9e-170 at 93% identity and 36% coverage) and *P. monodon* (GW327752.1, Expect = 1e-93 at 77% identity and 27% coverage). ORF 6 (frame 2+, bases 3055–4099 = 214 aa) gave a hit with a putative pol protein of the lepidopteran *Danaus plexippus* (EHJ74035.1, Expect = 2e-09 at 28% identity and 80% coverage) and an EST record for *P. vannamei* (CK591146.1, Expect = 1e-25 at 72% identity and 42% coverage). ORF 7 (frame +3, bases 4041–4967 = 308 aa) gave a BLASTp hit for an RNaseH in a pol-like protein from the mud crab *Scylla paramamosain* (GenBank: AEI88055.1, Expect = 3e-16 at 49% identity and 27% coverage), and a tBLASTn hit with EST records of *P. vannamei* (FE143676.1, Expect = 2e-131 at 97% identity and 61% coverage) and *P. monodon* (EE724334.1, Expect = 3e-79 at 72% identity and 50% coverage).

Although the other putative ORF (2 and 3) gave no significant hits using BLASTp, they gave strong hits with shrimp mRNA sequences using a tBLASTn search of crustacean EST sequence records at GenBank. For example, ORF 2 gave hits with *P. monodon* (GO072168.1, Expect = 6e-23) and *P. vannamei* (FE136984.1, Expect = 5e-17). Similarly, ORF3 gave hits with *P. vannamei* (CK591146.1, Expect = 2e-15) and *P. monodon* (GO072168.1, Expect = 5e-07).

### Phylogenetic analysis of reverse transcriptase

The pol-like proteins include three conserved domains for endonuclease/ exonuclease/ phosphatase, for reverse transcriptase and for RNaseH. Since the RT domain is somewhat more conserved in amino acid sequence than other retrotransposon sequences, it is usually selected for phylogenetic analysis and so it was here too.

**Figure 2:**
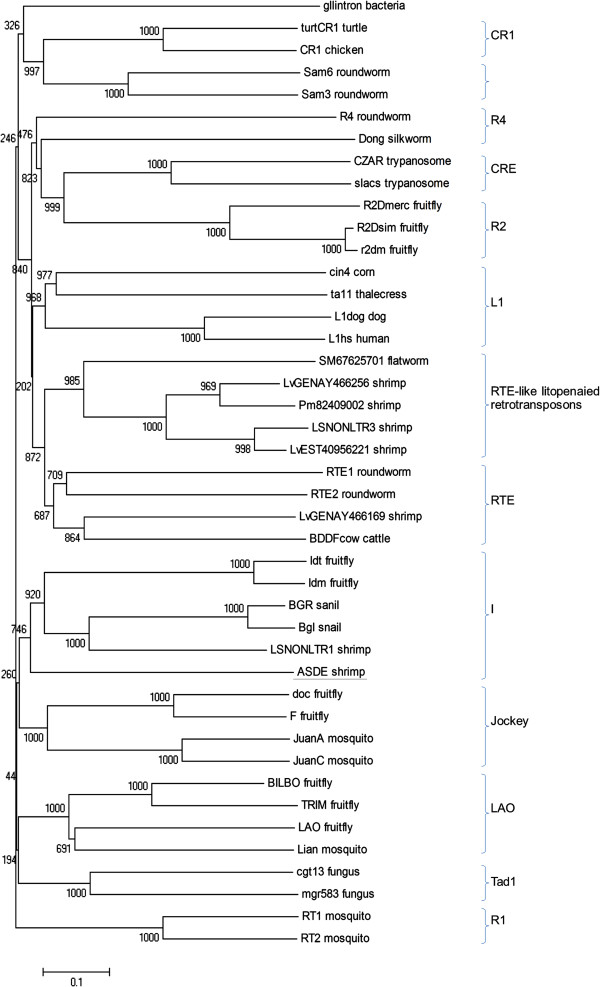
**Phylogenetic tree of RT sequences from non-LTR elements.** This comparison of reverse transcriptase (RT) domains of non-long terminal repeat (non-LTR) elements was constructed using default parameters for the neighbor-joining method of MEGA4 software. The numbers indicate the percentages of bootstrap support from 1000 replicates. The name of each non-LTR element and clade is given to the right. The accession numbers of elements represented are as follows: doc (X17551), F (M17214), JuanA (M95171), JuanC (M91082), Idt (M28878), Idm (M14954), BGR (X60372), BgI (EF413180.2), LOA (X60177), Lian (U87543), TRIM (X59239), BILBO (U73800), cgt13 (L76169), mgr583 (AF018033), Sam3 (U46668), Sam6 (Z82275), CR1 (U88211), TurtCR1 (AB005891), RT1 (M93690), RT2 (M93691), SM67625701 (CAJ00236), BDDFcow (M63452), RTE1 (AF025462), RTE2 (U58755), Dong (L08889), R4 (U29445), r2dm (X51967), R2Dsim (U13033), R2Dmerc (AF015685), cin4 (Y00086), ta11 (3047086), L1hs (U93574), L1dog (AB012223), slacs (X17078), CZAR (M62862), gIIintron (ZP_00604434), Pm82409002 (ABB73282), LSNONLTR1 (EU180975), LSNONLTR3 (EU180976), (LvEST40956221 (CK570639), LvGENAY466169 (AY466169) and LvGENAY466256 (AY466256).

Phylogenetic analysis was carried out based on the deduced amino acid sequence of the reverse transcriptase domain of ASDE and 12 major clades of non-LTR elements, including several previously reported from penaeid shrimp [2,4]. The tree was constructed using the neighbor-joining method and revealed that ASDE was most closely related to theI clade of such elements (Figure 2). This clade is represented by Idm and Idt elements from *Drosophila*[5], by BgI and BGR from the freshwater snail *Biomphalaria glabrata*[6] and by the non-LTR element LSNONLTR1 previously reported from *P. stylirostris*[2]. By contrast, the reverse transcriptase region of non-LTR retrotransposon Pm82409002 from *P. monodon* fell in clade RTE together with those of LSNONLTR3 from *P. stylirostris* and LvGENAY466169, LvGENAY466256 and LvEST 40956221 from *P. vannamei*[2].

**Figure 3:**
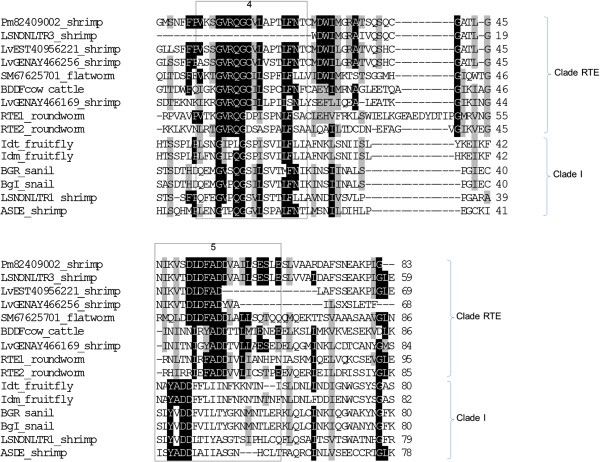
**Alignment of reverse transcriptase (RT) protein sequences.** Protein alignments of non-LTR retrotransposon clade I and RTE based on the reverse transcriptase (RT) sequence of the partial sequence of the reverse transcriptase domains 4–5 of Idt (M28878), Idm (M14954), BGR (X60372), BgI (EF413180.2), SM67625701 (CAJ00236), BDDFcow (M63452), RTE1 (AF025462), RTE2 (U58755), Pm82409002 (ABB73282), LSNONLTR1 (EU180975), LSNONLTR3 (EU180976), (LvEST40956221 (CK570639), LvGENAY466169 (AY466169) and LvGENAY466256 (AY466256). The numbers above the boxes indicate the domain. Amino acid similarity is represented with grey shading while identity is represented with black shading.

A multiple sequence alignment was carried out for reverse transcriptase domains 4–5 of ASDE and with other elements from clade RTE and clade I which contained the non-LTR retrotransposons from the penaied shrimp (Figure 3). The alignment revealed homology and conserved amino acid residues of two clades. The GQG conserved sequence of reverse transcriptase domain 4 of both clades were located at the same position while the 2 aspartic acid (DD) residues of domain 5 aligned differently for the two clades.

### PCR, RT-PCR, Southern blot and northern blot

**Figure 4:**
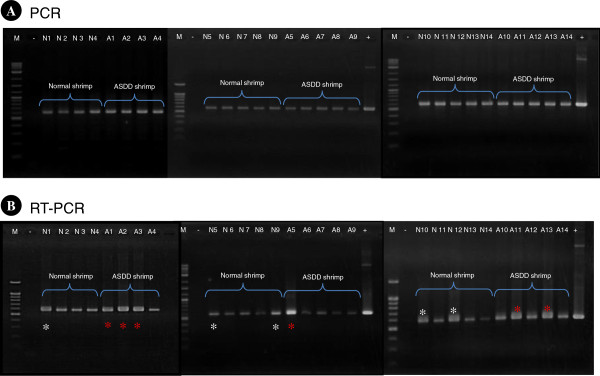
**PCR and RT-PCR detection of NLRS in ventral nerve cords.** Analysis of polymerase chain reaction (PCR) **(A)** and reverse transcriptase-polymerase chain reaction (RT-PCR) **(B)** products with primers specific to the NLRS sub-element in the ventral nerve cords of cultivated shrimp. Lane M, marker; -, negative control (sterile water); N, DNA and RNA extracts from normal shrimp; A, DNA and RNA extracts from ASDD shrimps and +, positive control (ASDE-plasmid). Asterisks (white for normal shrimp and red for ASDD shrimp) indicate strong reactions for NLRS RT-PCR products.

The PCR tests with DNA extracts and RT-PCR tests with DNase-treated RNA extracts from ASDD and normal shrimp revealed that all the shrimp were positive for the RT domain by both detection methods. The positive result by PCR suggested that the ASDE target sequence was present in shrimp DNA extracts while the positive result by RT-PCR indicated that an RNA form of ASDE (either reverse-transcriptase mRNA or “viral” genomic RNA) was also present in the extracts. For RT-PCR, strong reactions were observed in 5/14 normal shrimp and 6/14 ASDD shrimp (Figure 4). However, the mean density of the bands for the normal shrimp and the ASDD shrimp were not significantly different (P = 0.327).

**Figure 5:**
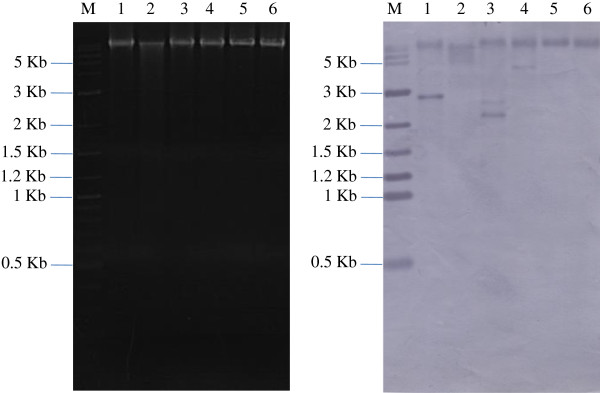
**Southern blot for detection of NLRS in host shrimp DNA.** RNase A-treated total DNA (5 μg) from ASDD shrimp was subjected to restriction enzyme digestion followed by agarose gel electrophoresis **(A)** and Southern blotting **(B)** using a DIG labeled probe specific for NLRS. M = marker; 1 = BamHI digest; 2 = NdeI digest; 3 = PstI digest; 4 = SalI digest; 5 = XbaI digest; 6 = XhoI digest.

Proposed incorporation of the ASDE sequence into the shrimp genome was supported by Southern blot analysis of the RNase-treated genomic DNA isolated from ASDD and normal shrimp digested with various restriction enzymes (BamHI, NdeI, PstI, SalI XbaI and XhoI) (Figure 5). These enzymes had 3, 1, 6, 1, 0 and 0 cutting sites, respectively, within the ASDE sequence. In all the tests, undigested genomic DNA gave positive hybridization with the DIG-labeled probe, and some enzymes (e.g., (XbaI and XhoI) gave no additional hybridization bands. However, digestion with the restriction enzymes BamHI, PstI and SalI gave one or more additional hybridization bands. For example, digestion with BamHI gave an additional positive band of approximately 2.7 kb, indicating that it contained the probe target sequence (Figure 5). The fact that this band was not present in the undigested DNA indicated that it was released from the total DNA by restriction enzyme digestion. Based on the diagram in Figure 1, the size of this fragment corresponded to an expected restriction enzyme fragment of 2.76 kb containing most of ORF5, all of ORF6 and a portion of ORF7. Unfortunately, attempted cloning and sequencing of the fragments represented by these additional bands from the restriction enzyme digests failed, and no chimeric sequences clearly composed of ASDE and host shrimp sequences were obtained.

PCR using primers to cover the whole ASDE sequence with DNA extract templates gave a PCR amplicon of approximately 4 kb only. However, sequencing of the cloned product revealed poor sequence identity with the 5 kb ASDE sequence obtained by 3′ and 5′ race (Additional file 3). However, both PCR and RT-PCR assays for the putative ORF of ASDE from the appropriate DNA and RNA templates from ASDD shrimp gave one product each for ORF 1 and another product each spanning ORFs 4 to 7 (4098 bp). No RT-PCR products were obtained for ORFs 2 and 3 in either reading direction. These results indicated that no single DNA or RNA target that contained all 7 of the ASDE ORF existed in ASDD shrimp. Attempts at northern blots with RNA extracts from ASDD shrimp failed to give the expected hybridization band at 4098 kb corresponding to the RT-PCR product covering ORFs 4 to 7 of ASDE as described above, probably because of too little transcript to be detected by the method used.

### *In situ* hybridization

**Figure 6:**
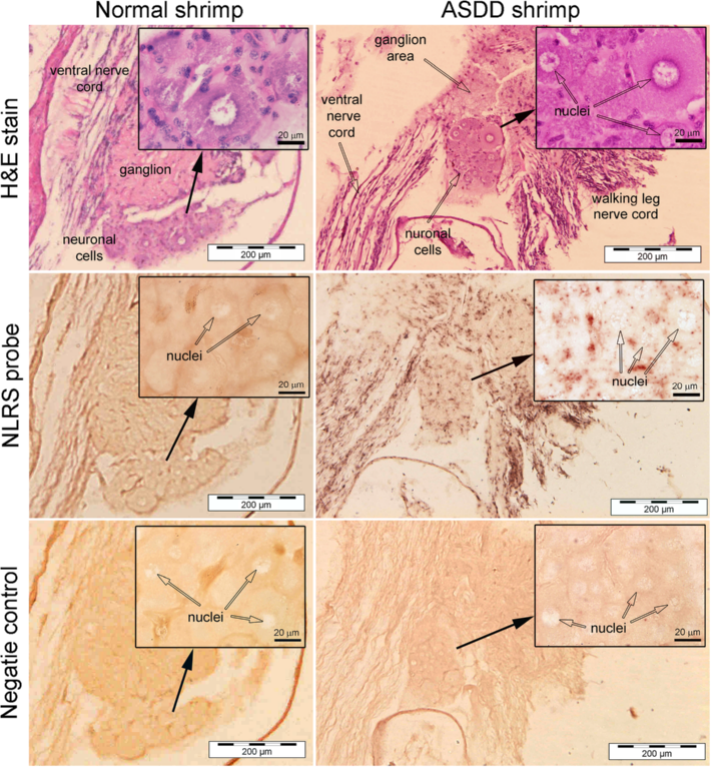
***In situ *hybridization tests.** Example of *in situ* hybridization results using a DIG-labeled NLRS probe with grossly normal (but NLRS positive) shrimp and with ASDD shrimp. Shown are contiguous sections each from normal and ASDD shrimp stained with H&E and subjected to the in situ hybridization procedure with and without the probe. Nerve cords of both were positive (dark staining) for NLRS (weak in normal shimp), but cytoplasm of neurons of ASDD shrimp only gave positive signals with the NLRS DIG-labeled probe.

The *in situ* hybridization results revealed a purple precipitate in all 10 normal and 10 ASDD shrimp tested and showed cytoplasmic localization in tubules and spheroids of the lymphoid organ, in hematopoietic tissue, in gills, in the ventral nerve cord, in globuli cells of the eyes and in connective tissue of the ovary and testis (data not shown). For most of these tissues, intensity of the positive reactions varied from specimen to specimen, but without significant differences between the two shrimp groups. However, for the abdominal ganglia, positive reactions were found in the cytoplasm of neurons of ASDD specimens only, not in specimens of normal shrimp (Figure 6).

**Figure 7:**
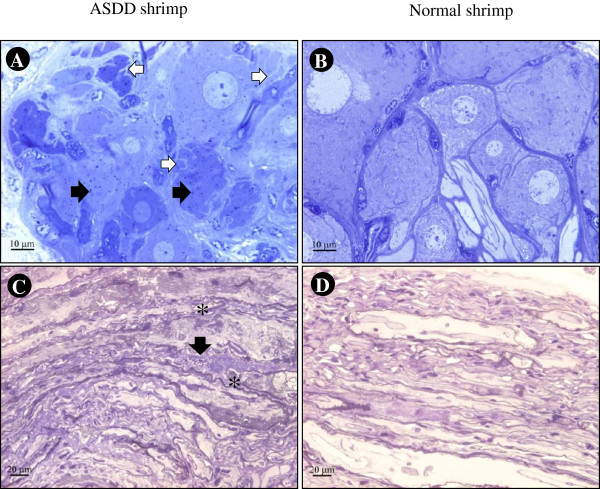
**Photomicrographs of ventral nerve cords.** Photomicrographs of toluidine-blue stained, semi-thin sections of the abdominal ganglia of normal and ASDD *P. vannamei* showing small inclusions (black arrows) and cracked neurons (white arrow) in the latter **(A)** when compared to normal neurons **(B)**. Irregular nerve fibers (asterisk) and small vacuoles (arrow) are seen in ASDD shrimp **(C)** but not in normal shrimp **(D)**.

As in previous results, semi-thin sections of the abdominal ventral nerve cord revealed an irregular pattern of fibre arrangement [1]. Here, additional anomalies were observed in the cytoplasm of neurons of the abdominal ganglia of ASDD shrimp. These included numerous small cytoplasmic inclusions accompanied in some neurons by fragmented cytoplasm. These features were not observed in the ganglia of normal shrimp (Figure 7).

### Transmission electron microscopy

**Figure 8:**
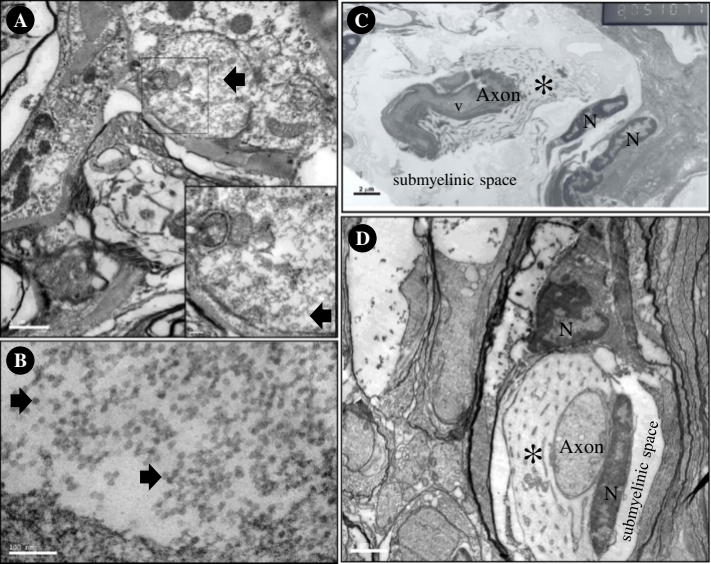
**Transmission electron micrographs.** Viral-like particles (approximately 20–22 nm) (arrow) can be seen in the cytoplasm of glial cells or neurons **(A, B)** of the abdominal ganglion of ASDD shrimp. The degenerated axon **(C)** is fragmented and dense and contains a vacuole (v), has a disorganized microtubular sheath (asterisk) and has a larger submyelinic space when compared that of a normal shrimp **(D)**. N = nucleus.

Under TEM, numerous viral-like, non-enveloped, icosahedral particles of 20–22 nm diameter were found in the cytoplasm and processes of glial cells, as in a previous report [1]. This study revealed additional abnormal ultrastructure of an axon from ASDD *P. vannamei* (Figure 8). Normal axons of penaeid shrimp are immediately surrounded by a microtubular sheath, inside a submyelinic space filled with an amorphous gel [7]. The microtubular sheath provides mechanical support for the axon, while the submyelinic space electrically insulates the axon, as well as provides some flexibility for it. Unlike normal features, the axons from ASDD *P. vananmei* became more-electron-dense, deformed, fragmented, and surrounded by disorganized and degraded microtubular sheaths. These degenerative changes were similar to those of degenerative axons observed in the crab *Ucides cordatus* following axotomy [8].

### Broodstock analysis and NLRS transmission to larvae

Table 1 shows results of the preliminary study of PCR and RT PCR of young and old broodstock. Four out of 5 of the old broodstock but only 1 out of 5 of the young broodstock were positive for NLRS by both one-step PCR and RT-PCR and the difference was statistically significant (P<0.01) by Chi-square analysis (SigmaStat software). In the young broodstock, the remaining specimens were either one-step, PCR-positive only, one-step RT PCR-positive only or both-negative. By nested PCR, however, all of the broodstock specimens were positive. By stereomicroscopy, mysis 3 from the young and old broodstock showed 30% and 50% prevalence of gross signs of ASDD (abdominal muscle deformity), respectively. Detection of NLRS in pooled mysis 3 from both broodstock sources were positive for NLRS by both 1-step PCR and 1-step RT-PCR.

**Table 1:** Detection of NLRS in broodstock and their larvae

		**PCR**	**RT PCR**
Four-week broodstock	Broodstock No. 1	+ve	+ve
	Broodstock No. 2	-ve	-ve
	Broodstock No. 3	-ve	-ve
	Broodstock No. 4	-ve	+ve
	Broodstock No. 5	+ve	-ve
	Pooled Mysis 3	+ve	+ve
Sixteen-week broodstock	Broodstock No. 1	+ve	+ve
	Broodstock No. 2	+ve	-ve
	Broodstock No. 3	+ve	+ve
	Broodstock No. 4	+ve	+ve
	Broodstock No. 5	+ve	+ve
	Pooled Mysis 3	+ve	+ve

Results of polymerase chain reaction (PCR) and reverse transcriptase polymerase chain reaction (RT-PCR) tests specific for the NLRS sequence using nucleic acid extracts from *Penaeus vannamei* broodstock pleopods and from pooled samples of their larvae (mysis 3). The broodstock specimens were utilized for either four or sixteen weeks in a hatchery.

In addition to this test, a random sample of 10 male shrimp was subjected to PCR assays for NLRS using DNA extracted from spermatophores and all gave positive results for NLRS by nested PCR. Since the shrimp sperm are spiked and do not have flagella, they contain no mitochondria and thus possess only chromosomal DNA. Thus, the results confirmed the presence of NLRS in shrimp genomic DNA and its heritability.

## Discussion

In this study, shotgun cloning followed by RACE led to the discovery of a new non-LTR retrotransposon-like element [3,9] named ASDE in *P. vannamei* showing gross signs of abdominal segment deformity disease (ASDD). The sequence contained an RT domain that shared homology with RT domains of non-LTR retrotransposable elements similar to long interspersed nuclear elements (LINE or L1-like elements) that constitute the most abundant classes of transposable elements in vertebrates [4]. L1 retrotransposons comprise 17% of the human genome and may play a role in modulating gene expression [10] even at the somatic level [11-13]. For example, human L1 was shown to alter the expression of neuronal gene Sox2 in the rat hippocampus neural stem cells *in vitro* and to influence neuronal somatic mosaicism in transgenic mice *in vivo*[14]. A similar phenomenon has been reported for *Drosophila*, where neural expression and mobility of retrotransposons can promote somatic neural diversity that may contribute to individual behavioural changes and/or neurological disorders [15]. By inference, it is possible that ASDE may alter the normal function of neurons in the abdominal ganglia of ASDD shrimp. Another example of a transposable element is the penelope retrotransposon-like element PEG11 (also called RTL1) that causes muscular hypertrophy in callipyge sheep by changing the expression of linked genes [16].

Normally, the reverse transcriptase domain is the conserved region most commonly found in retroelements and used to construct phylogenetic trees. Phylogenetic analysis of the deduced amino acid sequence of the reverse transcriptase domain of ASDE revealed homology to the I clade of non-LTR retrotransposons including Idt, Idm, BgI, BGR and LSNONLTR1 [2,5,6]. The transcriptionally active BgI element has been reported to be widely distributed in New World and Old World snails [6]. Intriguingly, BgI is flanked by 5′ and 3′ non-coding regions that show 80% identity to a schistsosome snail parasite and suggesting that it may have been acquired by horizontal transfer from the parasite to its snail hosts. Although the deduced amino acid sequence (frame 2) of ASDE gave the best BlastP hit (E=4e-^61^) at high coverage (96%) for the pol protein of a non-LTR retrotransposon from a snail, it shared only low (28%) amino acid identity, indicating that they are only distantly related.

A study of non-LTR retrotransposons of penaied shrimp [2] revealed that elements from *P. monodon*, *P. stylirostris* and *P. vannamei* were mainly grouped in an RTE clade (RTE-like litopenaied retrotransposons) while fewer elements were classified in clades CR1 and I. After several unanswered inquiries, we were unable to obtain sequences of 3 additional shrimp EST contigs (LVESTcontig7+1, LVESTcontig11+1 and LVESTcontig12-3-2) of *P. vannamei*[2] for inclusion in our phylogenic tree. However, we can assume that they would have fallen in our clades for CRE1 (LVESTcontig12-3-2) and RTE-like (LVESTcontig11+1) and LVESTcontig7+1 as they did in the previous publication [2].

In summary, none of the previously reported non-LTR elements from shrimp fell in the same clade (I) as NLRS and none have been reported to be associated with particular shrimp phenotypes. This is true also for insects, where most of the work on retrotransposale elements has been focused on evolutionarly analysis. On the other hand, it has been reported previously that environmental stress can increase expression of such elements in shrimp [17,18].

Curiously, putative ORF 1 of the ASDE sequence showed high homology to a GIY-YIG catalytic endonuclease domain of Penelope-like transposons (e.g., GenBank: EFN65003.1 for the ant *Camponotus floridanus* Expect = 5e-11) that belong to a clade of retrovirus-like transposable elements phylogenetically separate from the non-LTR transposons [3]. This initially suggested that ASDE might be a chimeric construction of two different types of retro-transposable elements. However, the failure to amplify a single 5 kb transcript containing the GIY-YIG sequence together with the pol-protein-like sequence suggested that the GIY-YIG sequence and the pol-protein-like sequence were not linked as appeared in the originally concatenated ASDE sequence obtained by 3′/5′ RACE. This was confirmed by sequence comparison between ASDE and a 4 kb fragment amplified by PCR using primers designed from the 5′ and 3′ ends of ASDE. The sequence of the amplified fragment showed high homology to ASDE at both ends but contained no GIY-YIG element. In addition, good homology to the non-LTR region of ASDE was found only for the 3′ end of the RT portion and the adjacent downstream RHaseH. Altogether, the data indicated that the 4 kb amplicon arose from a different location in the host shrimp genome where only a portion of the non-LTR region of ASDE was duplicated. Fortunately, the region with high homology to the ASDE reverse transcriptase (RT) domain did not match well with the target we used to detect the RT by PCR and RT-PCR, nor with the sequence of the corresponding cDNA probe that was used for its detection in blots and *in situ* hybridization assays. From this point onward, we will refer to the GIY-YIG Penelope-like sub-element of ASDE as PENS (GenBank: KC179708) and the non-LTR-like sub-element as NLRS (GenBank: KC179708).

NLRS is clearly not a retrovirus [19] because its genome structure does not include (in order) a gag protein, a pol region containing a protease (PR), a reverse transcriptase (RT) and an RNaseH, and finally an envelope (env) protein, and because its RT sequence is phylogenetically related to those of non-long terminal repeat (non-LTR) retrotransposons. In addition, it fits in the I clade of non-LTR elements because of its RT domain homology (Figure 2) and because the RT domain is followed by an RNAseH domain [20]. Of the retrovirus-like retrotransposons known, viral families have been described only for the long terminal repeat (LTR) retrotransposon types Ty1-copia (Family *Pseudoviridae*) [21] and types Bel and Ty3-gypsy (Famly *Metaviridae*) [22] whose genome structures contain (in addition to RT and RNaseH) a gag protein, a protease (PR) and an integrase (IN) [3], all three of which are absent from the ASDE sequence. Although it is possible that extension of the ASDE sequence on the 3′ side of its RNaseH sequence might eventually reveal IN and env sequences, the 5′ side of the RT sequence contains neither PR or IN sequences.

This information, together with the fact that putative ORF 4 to 7 could be amplified from both DNA and RNA templates of ASDD shrimp while the complete 5 kb of ASDE could not, and the fact that chromosomal DNA could hybridize with the RT probe specific for NLRS suggested that NLRS was a host chromosomal element. This would be consistent with the characteristics of non-LTR retrotransposable elements reported from other eukaryotes where they are believed to be transmitted exclusively vertically [20]. This inferred that NLRS was not a representative of any retrotransposable construct with a single RNA genome that might be capable of producing viral-like particles such as those seen in the families *Retroviridae*, *Metaviridae* or *Pseudoviridae*. This, in turn, suggested that the viral-like particles in the neuronal cells positive with the ASDE-RT probe did not arise directly from the ASDE element, leaving their origin open to speculation.

The situation regarding the causal role of NLRS in ASDD was complicated by our fortuitous discovery of the unconnected PENS in the ASDE construct. Since a Penelope transposon has been shown to be causally involved in muscular hypertrophy in callipyge sheep [16], it is reasonable to ask whether the similar PENS from shrimp might also be associated with ASDD. To answer this question, it would be necessary to compare the expression of both of these ASDE sub-elements in the neuronal cells that show pathology in ASDD shrimp and in aging broodstock together with their ASDD offspring. If the affected neuronal cells of only ASDD shrimp were positive for the NLRS probe and negative for the PENS probe, it would support the proposal for unique association of NLRS with ASDD. If both probes were positive in neuronal cells of only ASDD shrimp while normal shrimp showed only negative results for the both probes in neuronal cells, it would indicate that both elements were associated with ASDD. Other possible results would lead to different conclusions. For the first two outcomes, elimination of NLRS from breeding stocks would eliminate ASDD, whether PENS was an associated, component cause of the disease or not.

In summary, the preceding information indicates that NLRS has structural similarity and homology to vertically-transmitted non-LTR elements, that an NLRS probe in southern blots shows positive hybridization with total shrimp DNA and with an expected 2.76 kb BamHI digestion fragment released from it, and that a full-length transcript of NLRS is present in spermatophore DNA of both normal and ASDD shrimp. Thus, NLRS can be transmitted from grossly normal parental shrimp to their offspring, but anecdotal information from shrimp farmers indicates that althgough muscle deformity persists, it does not increase in prevalence with cultivation time (i.e., no horizontal transmission). The occurrence of a homologous sequence in the *P. monodon* EST database suggests that this element may be an ancient acquisition in penaeid shrimp and that homologues may occur also in other species of the genus.

What remains to be explained is why there tends to be higher expression of NLRS transcripts in well-used female broodstock of *P.vannamei* than in newly used female broodstock and how higher expression can lead to a higher proportion of deformed offspring. The tools we have developed can be used to quantify NLRS transcripts in young and old broodstock in response to stress from various environmental changes that occur in shrimp hatcheries [23]. They can also be used to study the consequences of increased expression of NLRS transcripts. Judging from what has been reported previously for increased expression of Line-1 elements in neuronal cells of vertebrates and *Drosophila*[11-13], the effects will probably be complex and involve NLRS-mediated alteration of neuronal-cell gene expression leading ultimately to muscle deformity. How this effect could be carried over from the broodstock to development of the offspring is an intriguing question, especially since it results in a specific rather than random type of abnormality.

## Conclusions

Although both normal and ASDD shrimp gave positive results for the ASDE sub-element NLRS by PCR and RT-PCR, *in situ* hybridization assays for NLRS gave positive signals only in the cytoplasm of neurons of ASDD shrimp, and this was accompanied by neuronal degeneration in the abdominal ganglia. There was a positive relationship between the long-term use of female brooders, the increased expression of NLRS RNA transcripts and an increased prevalence of ASDD in their progeny. Thus, we hypothesize that NLRS transcripts are up-regulated by stress in broodstock females and that this causes ASDD in their offspring by a currently unknown mechanism. The possibility of a causal, co-involvement with PENS should also be investigated.

Our recommendations for immediate control of ASDD include avoidance of long-term broodstock use and use of PCR screening to exclude broodstock that give positive reactions for NLRS. If the latter is not possible, RT-PCR could be used to exclude female broodstock that give 1-step RT-PCR reactions for NLRS. Ultimately, if NLRS-free stocks can be identified, the objective would be to eliminate NLRS from breeding stocks. The sequence information and PCR tools provided here will help in this work and in further study of NLRS and PENS. They will also be useful for examination of other cultivated shrimp species should they exhibit similar muscular deformity.

## Methods

### Samples of farmed ASDD *P. vannamei* juveniles and tissue preparation

ASDD and normal *P. vannamei* juveniles (4 months in culture) from commercial ponds were arbitrarily sampled. The Ethical Principles and Guidelines for the Use of Animals of the National Research Council of Thailand (1999) apply to vertebrates only and there is no official standard for invertebrates. Nor could we find any regulations regarding shrimp in the national legislation of any country. However, we did find guidelines (not regulations) of the Australian, New South Wales government for the humane harvesting of fish and shellfish that included guidelines for large crustaceans such as lobsters and crabs (but not shrimp) (<http://www.dpi.nsw.gov.au/agriculture/livestock/animal-welfare/general/fish/shellfish>; link reconfirmed on 16 February 2013). These include recommendations regarding the transport of the crabs and lobsters and to their laboratory maintenance. We followed these guidelines for transport and maintenance and we processed the shrimp for histological analysis or for killing at the end of an experiment using the saltwater/ice slurry method recommended.

From each group, ten shrimp were sectioned sagittally in the thoracic and abdominal parts [24] and fixed with Davidson’s fixative for *in situ* hybridization (ISH). Ventral nerve cords were isolated from five individual shrimp in each group and fixed in 4% glutaraldehyde in 0.15 M Millonig’s phosphate buffer for transmission electron microscopy (TEM). Fourteen nerve cords of ASDD shrimp were pooled and frozen in liquid nitrogen for nucleic acid extraction and cloning.

### Total nucleic acid extraction

Frozen ventral nerve cords from 14 shrimp showing gross signs of ASDD (i.e., abdominal muscle deformity) were ground to powder in the presence of liquid nitrogen. The powder (approx. 300 mg) was re-suspended in 200 μl of TN buffer (0.02 M Tris–HCl, 0.4 M NaCl, pH 7.4), before centrifugation at 3,120 ×g for 30 min. The supernatant was collected and the process was repeated. The pooled supernatants were ultracentrifuged at 300,000 × g for 2 h at 4°C (Beckman XL-90 Ultracentrifuge, Fullerton, CA). The resulting pellet was dissolved with TN buffer and mixed with 500 μl of TRIzol reagent (Invitrogen™, Carlsbad, CA) before nucleic acid extraction following the Trizol reagent protocol. After 500 μl of chloroform was added, the RNA fraction was removed. Then ethanol was added to the organic phase and interphase for DNA extraction according to the kit protocol. The RNA and DNA preparations obtained from both extraction processes were pooled as a total nucleic acid stock and stored at −20°C. The purity of the pooled nucleic acid sample was determined by measuring the ratio of OD_260nm/280nm._ The total nucleic acid stock was used for random-prime cDNA synthesis, followed by cloning and sequence analysis. It was also used for subsequent treatments with RNase and DNase followed by Southern blot, Northern blot, PCR and RT-PCR methods.

### cDNA synthesis, cloning and sequence analysis

To cover the possibility of both RNA and DNA viruses, the total nucleic acid stock (1 μg total DNA/RNA mix) was subjected to the cDNA synthesis protocol described in the Marathon™ cDNA Amplification kit manual (BD Biosciences, San Jose, CA). The final cDNA plus original DNA mix was randomly cloned using a pGEM-T Easy vector system (Promega, Madison, WI) and transformed into *E. coli*. DNA extracts from clones containing plasmids with inserts were subjected first to southern blot hybridization using a random hexamer prime DIG-labeled normal shrimp DNA probe to eliminate clones containing shrimp sequences [25]. Clones giving negative or weak hybridization results were subjected to sequencing (Macrogen Inc., Korea). Basic local alignment search tool (BLAST) analysis from the National Center for Biotechnology Information (NCBI) was used to analyze the sequences. Deduced amino acid sequences were aligned using the multiple alignment ClustalW2 program (http://www.ebi.ac.uk/Tools/msa/clustalw2). Phylogenetic trees were generated by the neighbor-joining method using 1,000 bootstrap replicates by MEGA4 software with default parameters [26] and following the general approach previously used or comparison of shrimp retrotransponson-like elements [2].

### Rapid amplification of cDNA ends (RACE)

Rapid Amplification of cDNA Ends (RACE) technique was used to extend the sequence from the RNA of ASDE using the SMART™ RACE cDNA Amplification Kit (Clontech, Mountain View, CA) according to the manufacturer’s instructions. Total RNA derived from the total nucleic acid extract of ASDD shrimp (see section on total nucleic acid extraction) was used as a template for cDNA synthesis together with three sets of primers in separate experiments. The pairs of gene specific primers for 5′ and 3′ RACE are shown in Table 2 and the reaction mixtures used were those provided with the kit. The first PCR cycling conditions were 5 cycles of 94°C for 30 seconds and 72°C for 3 minutes followed by 5 cycles of 94°C for 30 seconds, 70°C for 30 seconds and 72°C for 3 minutes followed by 25 cycles of 94°C for 30 seconds, 68°C for 30 seconds an 72°C for 3 minutes. In the nested PCR reaction, the primary PCR product was used as the template. Conditions for the nested PCR amplification were 94°C for 15 seconds followed by 30 cycles of 94°C for 30 seconds, 68°C for 30 seconds and 72°C for 3 minutes and an additional extension at 72°C for 7 minutes. After agarose gel electrophoresis of the PCR product, bands were purified from the agarose gel, cloned using a TA cloning kit (pGEM T-easy) and sequenced in both directions to achieve 100% overlap. If mismatched bases appeared, another round of sequencing was carried out to obtain a consensus sequence for each clone. The process yielded a set of clones (Table 3) with overlapping sequences that could be assembled into one uninterrupted sequence of 5052 bp using the same methods as described in the preceding section of the methods.

**Table 2:** PCR and RT-PCR primers used

**Primer name (amplicon length)**	**Primer sequence (5′ to 3′)**
**PCR/ RT-PCR (600)**	
Reverse transcriptase	
Forward primer	AGCTGGAAACAAGCCACAAT
Reverse primer	ACAACACTCTTCGGACACCA
**ORF1 (388)**	
Forward primer	CGAGACAAGAAGCAGTTGAAAA
Reverse primer	TGGGAGCTGGTAAATCTCCT
**ORF2 (249)**	
Forward primer	TCCAGCTTCATCTACATGATCTACC
Reverse primer	GCATATACTTTGGAAAAAGTGAAAGTC
**ORF3 (276)**	
Forward primer	GGCGACTTTCACTTTTTCCA
Reverse primer	GCTCGTTTTCGGTTTGGAG
**ORF4 (388)**	
Forward primer	TGAAGAAAGCAGTGCAGGTC
Reverse primer	GCTTCAGATGCCCCAGTG
**ORF5 (2146)**	
Forward primer	TGGCACACAATCTAAGCATTC
Reverse primer	CATTCCAGATCCATACCCTGT
**ORF6 (644)**	
Forward primer	TGGAGATCCAATACCTGCTTG
Reverse primer	TCAATGACCCTTTGTGCTTG
**ORF7 (916)**	
Forward primer	TGAGAAAAGATCAATATTCCATGC
Reverse primer	TTTGAACTGCATGCTTACCG
**ORF1-7 (4941)**	
Forward primer	CGGCCTGTGCAGACTAATG
Reverse primer	TTTGAACTGCATGCTTACCG
**ORF4-7 (4114)**	
Forward primer	TGAAGAAAGCAGTGCAGGTC
Reverse primer	TTTGAACTGCATGCTTACCG
**ASDE**	
Forward primer	CGGCCTGTGCAGACTAATG
Reverse primer	AGTTAGATTTGCGAAGTCATGC
**5′RACE**	
FirstRt_ASDEF1	TGCCTGACCCGTTTCCACAACTCT
NestRt_ASDEF1	GCTGCCTGGTTTATGGCCTGGATTAG
FirstRt_ASDEF2	GCTGCCTGGTTTATGGCCTGGATTAG
NestRt_ASDEF2	CTATGTGTGCATTGAAGTCTACCCCTAT
FirstRt_ASDEF3	GAAACGGGACGAGACTTCAACACCA
NestRt_ASDEF3	TGCAATTGTCGCTCGGAGGTTACTG
**3′RACE**	
FirstLt_ASDER1	CCGTGGTTGTCTTCCTTGACCTGGA
NestLt_ASDER1	TCAGCGTTGTCTGAACCTGGTGTCC
FirstLt_ASDER2	TTTGCGGACAATTCCTGGCTCACAC
NestLt_ASDER2	CCATGCCTATCCTAAAGGCACAAGCACA
FirstLt_ASDER3	CCACTGGCCCTTCCTCCAAATACAA
NestLt_ASDER3	CCGCGGTAAGCATGCAGTTCAAAG

**Table 3:** ASDE plasmid clones

**Name**	**Position**	**ORF contained**	**Length**
ASDE clone 1 (5′ Race)	1 - 1539	ORF 1, 2, 3, 4, 5	1539 bp
ASDE clone 2 (5′ Race)	1540 - 2029	ORF 5	490 bp
ASDE clone 4 (Original clone)	2030 - 3345	ORF 5	1316 bp
ASDE clone 5 (3′ Race)	3346 - 4161	ORF 5, 6, 7	816 bp
ASDE clone 6 (3′ Race)	4162 - 5052	ORF 7	891 bp

Details of plasmid clones (pGEMT-easy) containing fragments of the ASDE sequence.

### NLRS PCR, RT-PCR and DIG probe labelling

A specific pair or primers was designed for the detection of the reverse transcriptase domain of ASDE (Table 2). PCR reactions were performed in 25 μl of 1x PCR buffer, 1.5 mM MgCl_2_, 0.5 μM each forward and reverse primer, 0.2 mM each dNTPs and 2.5 units *Taq* DNA polymerase and 100 ng DNA template according to the following protocol: initial denaturation at 94°C for 5 min followed by 30 cycles of denaturation at 94°C for 30 sec, annealing at 50°C for 30 sec and elongation at 72°C for 60 sec plus an additional elongation step at 72°C for 7 min. RT-PCR was carried out in a single reaction using the SuperScript™ III one-step RT-PCR system with a *Platinum®Taq* DNA polymerase kit (InvitrogenTM, Carlsbad, CA). The protocol was the same as for the PCR reaction above except for the use of 100 ng RNA template and the addition of an RT step at 50°C or 30 min prior to the PCR step. Following this, aliquots of the PCR and RT-PCR reaction mixtures were subjected to separation by 1.2% agarose gel electrophoresis followed by staining with ethidium bromide and visualization of a 600 bp amplicon by UV transillumination.

A specific digoxygenin-dUTP probe (600 bp) was prepared (kit from Roche Diagnostics GmbH, Mannheim, Germany) to detect the presence of the non-long terminal repeat transposon-like sub-element (NLRS) in the ASDE sequence by Southern blot, Northern blot and *in situ* hybridization (ISH). The primers for the labelling reaction were the same as those given above for the reverse transcriptase (RT) domain of NLRS (Table 2) and were used with a plasmid containing an ASDE insert that included the RT sequence (Table 3). The same primers were used to detect NLRS by either PCR or RT-PCR. The DIG-labeled probe was purified using a QIAquick PCR purification kit (QIAGEN, Hilde, Germany) according to the manufacturer’s instructions. The probe concentration was determined by measuring OD_260_ and its specificity was verified by dot blot hybridization. The probe was stored at −20°C.

### Southern blotting

The total nucleic acid extract stock was treated with RNaseA (Geneaid, Tao-Yuan, Taiwan) at 37°C for 1 h and digested with *Eco*R I restriction enzyme (New England Biolabs, Beverly, MA) 37°C for 1 h. Following electrophoresis, gels were denatured and neutralized and bands were transferred to a nylon membrane (Roche Diagnostics GmbH, Mannheim, Germany). The hybridization protocol followed that described by Sambrook *et al*., (1989) [27]. Detection was performed using the same probe as in the *in situ* hybridization reactions described above and with an alkaline phosphatase-conjugated anti-DIG antibody (Roche Diagnostics GmbH, Mannheim, Germany) at 1:5,000 in blocking solution. The hybridization signal was developed by incubating the membrane in substrate buffer containing NBT/BCIP solution (Roche Diagnostics GmbH, Mannheim, Germany) in a dark box. The reaction was terminated by washing with distilled water followed by air-drying.

### Northern blotting

RNA from ASDD shrimp was treated with DNase I (New England Biolabs, Ipswich, MA). Then RNA was separated by electrophoresis on a 1.2% agarose gel and transferred onto a nylon membrane (Roche Diagnostics GmbH, Mannheim, Germany). The hybridization protocol was carried out as described by Sambrook *et al*., (1989) [27]. The membrane was hybridized overnight at 68°C with a digoxigenin-labeled ASDE probe. After incubation with an alkaline phosphatase-conjugated anti-DIG antibody (Roche Diagnostics GmbH, Mannheim, Germany), the hybridization signal was developed with NBT/BCIP solution (Roche Diagnostics GmbH, Mannheim, Germany). The reaction was terminated by washing with distilled water.

### PCR and RT-PCR screening of shrimp

Nucleic acids were extracted individually from ventral nerve cords of normal shrimp and shrimp exhibiting gross signs of ASDD. For DNA, individual ventral nerve cords were homogenized in 500 μl DNA lysis buffer (50 mM Tris HCl, pH 9.0, 100 mM EDTA, pH 8.0, 50 mM NaCl, 2% SDS, 1 μg/ml) and the homogenate was subjected to DNA extraction as previously described [27]. The concentration of the extracted DNA was measured at OD_260nm_.

RNA from individual ventral nerve cords was extracted using 1 ml Trizol-reagent (Invitrogen™, Carlsbad, CA) following the reagent manual. The concentration of the extracted RNA was measured at OD_260nm_ and the purity by using the ratio of OD_260/280nm_. To remove any DNA contamination, the extracted RNA was digested with DNase I (New England Biolabs, Beverly, MA) at 37°C for 30 min. Then the RNA was extracted again using Trizol-reagent by the same process.

### *In situ* hybridization

*In situ* hybridization was carried out according to the methods described by Sritunyalucksana *et al*. (2006) [28]. Briefly, the fixed tissues were processed through paraffin embedding, sectioned at 5 μm thickness, treated with Proteinase K, and post-fixed with ice cold formaldehyde. The sections were pre-hybridized in 4x SSC containing 50% deionized formamide and hybridized with the dioxygenin (DIG)-labeled probe. The sections were then incubated with anti-DIG antibody, and color reactions were developed using NBT/BCIP substrate (Roche Diagnostics GmbH, Mannheim, Germany) combined with Bismarck brown Y (Sigma-Aldrich, St. Louis, MO) counterstaining. Negative control sections were treated identically, but without DIG-probe.

### Electron microscopy

After fixing at 4°C overnight, the ventral nerve cords were washed with 0.15 M Millonig’s phosphate buffer and post-fixed with 1% osmium tetroxide in 0.15 M Millonig’s phosphate at 4°C for 1 h. They were dehydrated through an ethanol series followed by propylene oxide prior to embedding in Epon 812. Semi-thin sections were stained with toluidine blue and observed by light microscopy (LM). Ultrathin sections were stained with uranyl acetate and lead citrate before observation by TEM.

### Broodstock analysis and ASDE transmission to larvae

Two groups of mated female *P. vannamei* broodstock (60-65 g body weight ) were employed in this study. One group (young) had been eyestalk ablated and kept in a hatchery for intermittent spawning for four weeks and the other (old) for sixteen weeks. The two groups were kept in separated round concrete tanks, 4 m in diameter, 0.5 m-deep and containing clean, sand-filtered and ozonized, seawater (30–32 ppt, pH 7.8, alkalinity 150 ppm, at 28–30°C), with adequate aeration (oxygen >6 ppm). The water was monitored daily to control total ammonia and nitrite levels (<0.1 ppm). The broodstock were provided with live specific pathogen-free polychaetes (*Perinereis nuntia*) as feed at 3% BW ration. After spawning and hatching, the nauplii were raised to mysis 3 when the percentage of ASDD shrimp was determined by assessing anatomical distortion microscopically. After spawning, pleopods of individual broodstock were collected for DNA and RNA extraction and tested for ASDE by PCR and RT-PCR. Pooled mysis 3 samples derived from each broodstock specimen from the four-week group and the sixteen-week group were also tested for ASDE by PCR and RT PCR.

## Competing interests

The authors declare that they have no competing interests.

## Authors’ contributions

WS contributed to the design of the experiments, conducted most of the experimental work, analysed the results and drafted the manuscript. BP assisted in sample collection and in interpretation of the results. PP carried out the transmission electron microscopy. TWF assisted with *in situ* hybridization analysis, sequence analysis and drafting of the manuscript. BW contributed to the study concept, supervision of the work and drafting of the manuscript. All authors read and approved the final manuscript.

## Supplementary Material

Additional file 1 **MGID sequences.** After submission of the manuscript, the URL http://www.marinegenomics.org lapsed. However, we have the sequences for MGID512728 and MGID126456 and they are reproduced here in FASTA format. Click here for file

Additional file 2 **ORFs of ASDE.** The 7 putative open reading frames (ORFs) of ASDE 5052 bp corresponding to deduced amino acids. Click here for file

Additional file 3 **Alignment of ASDE and a PCR amplicon sequence.** Clustral alignment of the ASDE sequence (5052 bp) and a clone of the 4091 bp PCR amplicon obtained using primers designed to amplify the whole 5052 sequence from a total DNA template extracted from ASDD shrimp. The location of the reverse transcriptase target for RT-PCR, PCR and the hybridization probe is indicated in grey outline and the primer sequences are underlined. Click here for file
