# Supplementary material for: Discovery and partial characterization of a non-LTR retrotransposon that may be associated with abdominal segment deformity disease (ASDD) in the whiteleg shrimp Penaeus (Litopenaeus) vannamei
Source: BMC Vet Res. 2013 Sep 30;9:189. doi: 10.1186/1746-6148-9-189 (PMC3849965; doi:10.1186/1746-6148-9-189)
Supplement: Additional file 1 — MGID sequences. After submission of the manuscript, the URL http://www.marinegenomics.org lapsed. However, we have the sequences for MGID512728 and MGID126456 and they are reproduced here in FASTA format. [file 1746-6148-9-189-S1.docx]

**Additional file 1: MGID sequences.** After submission of the manuscript, the URL <www.marinegenomics.org> lapsed. However, we have the sequences for MGID512728 and MGID126456 and they are reproduced here in FASTA format.

>MGID512728

ACCATCTTCTCATCTGTTTTACCCAGGCAGCTCAGAAGAGAGATGGGACGGTATTTGCCAGGCTCCTTTGGTTTTGGGATGGGAACTATTGTGGTTTGTTTCCAGCTCTGGGGCACCGTGGCGGTTTGCCAGGATTTGTTGATAAGTTGCAAGAATGCAAGCTCTCCTGCCAGACCTAAATGCGATATGATGGGGTAGGAGATCCCATCAGATCCCGGTGCTGATCCAGAGCTGGTTTTGTATGACTTTCTTAGTTCCCTAAGAGAGAACAAGATCTGTTTCATCAGCTTCGAGTGCCTTGTCTCTTATGAGAGCAAATCTTTCTGGATTTAAGGTTTCTTGTTTTTCTCTTATCATTGGAGGCAGATTGTTGGTGTTGGTTCTGGCTGAGAACTCAAGCACCAATCTGTTAGCCTTTGATTGTGGGTCATGATGAGTGCATTTCGGGGCTTGGCGGCTTGGCGCTTGCCTGACCCGTTTCCACAACTCTGTAAGGCTGGTCTGGTGCCCAAAAGTTTGGCACCATTCCAGCCATTTTTCCTGCCTTACTCTGTTGGCAGTTTCCGTGGCATCCATGACAGCTTCCCTCAACGGGGCCAGATTGTCGGGAGATCTTTGCCGTCGGAAGTTTTTTCTACACATATTAACCCTGTGGTTGACCTCTTTGATCTCGTCATTATAGT

>MGID126456

TCCATTCCCTGGATTTTCAGACTTGTGCCTTGAACTCTCTGTCTCAAAGCCATGGCCTTGGGATTTGGGCTGCAGAGATCTTTAGTCCTGTCCTGCAACACTCCTCGGATACCAAGTCCAGACAACGCTGGGCTTTGTTTAGGCTGTGTGGTCCAGTGGAGATGATAGCAAGATCGTCTGCATAGGAGATAATCTTGCACCCCACTGGGAGGTTTATGTTGAGGATACAGGACATTAGTGTGTTAAAAAGGGCTGGACTGAGAACCCCACCCTGGGGNGGTCCCATTTTCCTANTNGGCATGTGCTGTGATAGGTGACCTTGGAATTTGACTCTGGCTGTTCTATTCGTAAAGTAGTCACCTATCCAAGCCAAGAGCTTACCTCTGATTCCTTTTTGGATCAGGCTCTCCTGAATGGCAAGAGGACTTGCCAATTCGAAAGCCTTCTCCAGGTCAAGGAATACTGCCACAGATGCGCCCGTGTTTATTGTGCTCAAGAGTGTGGCTATGCTGTGCGCTGTGCTCATGCCCCTGGTGAACCGTGGAGGTGTTCGTGTGGGGCTCCCA
