# Supplementary material for: Discovery and partial characterization of a non-LTR retrotransposon that may be associated with abdominal segment deformity disease (ASDD) in the whiteleg shrimp Penaeus (Litopenaeus) vannamei
Source: BMC Vet Res. 2013 Sep 30;9:189. doi: 10.1186/1746-6148-9-189 (PMC3849965; doi:10.1186/1746-6148-9-189)
Supplement: Additional file 2 — ORFs of ASDE. The 7 putative open reading frames (ORFs) of ASDE 5052 bp corresponding to deduced amino acids. [file 1746-6148-9-189-S2.doc]

1 CTGTCGGGGAGCCCGGCCTGTGCAGACTAATGTCGACTCGATCAACGTGTATCTACTAGTGCTGACGCGACAGAGCTTAGTCCTTACCCA
 1 GACAGCCCCTCGGGCCGGACACGTCTGATTACAGCTGAGCTAGTTGCACATAGATGATCACGACTGCGCTGTCTCGAATCAGGAATGGGT

91 CCGCCGTGCCCAGCCACAGCAGTAACCTCCGAGCGACAATTGCAACTTCTCGCGCCTGGGCGGGGAGCGAACCGCCGACCCCTCGGATGA

     91 GGCGGCACGGGTCGGTGTCGTCATTGGAGGCTCGCTGTTAACGTTGAAGAGCGCGGACCCGCCCCTCGCTTGGCGGCTGGGGAGCCTACT

    181 GAGGACGGCACGTTACCACTGTACTAGCCTGGAGGCTACGTAGCATATATATACTACATGGGAGCTGGTAAATCTCCTGACGACTGTGTC
    181 CTCCTGCCGTGCAATGGTGACATGATCGGACCTCCGATGCATCGTATATATATGATGTACCCTCGACCATTTAGAGGACTGCTGACACAG
 -2fr ··M··P··A··P··L··D··G··S··S··Q··T··

     271 CTCGCACTCGTTACGCGCATAATTGCAGCTGCGACCTTGGATACTTTAAACCTGCCTGATGCGGTGTTCATATTCTTTTCTGTCGCGATG
     271 GAGCGTGAGCAATGCGCGTATTAACGTCGACGCTGGAACCTATGAAATTTGGACGGACTACGCCACAAGTATAAGAAAAGACAGCGCTAC
 -2fr R··A··S··T··V··R··M··I··A··A··A··V··K··S··V··K··F··R··G··S··A··T··N··M··N··K··E··T··A··I··

+3fr M··S··S··F··I

     361 TATGCAGCTTCCATGACCTTCCTTTTATGTTTACTCACTCTTTCATGGATTAATTTGGCTTCCTTCCAGTTCGGTAGATGTCCAGCTTCA
     361 ATACGTCGAAGGTACTGGAAGGAAAATACAAATGAGTGAGAAAGTACCTAATTAAACCGAAGGAAGGTCAAGCCATCTACAGGTCGAAGT
 -2fr Y··A··A··E··M··V··K··R··K··H··K··S··V··R··E··H··I··L··K··A··E··K··W··N··P··L··H··G··A··E··

·

+3fr ·Y··M··I··Y··H··G··I··G··S··P··V··V··T··D··V··G··S··L··F··D··D··P··G··V··E··A··S··S··R··F·

     451 TCTACATGATCTACCATGGCATTGGAAGTCCTGTGGTGACGGACGTCGGCTCGCTGTTCGATGATCCTGGTGTTGAAGCCTCGTCCCGTT
     451 AGATGTACTAGATGGTACCGTAACCTTCAGGACACCACTGCCTGCAGCCGAGCGACAAGCTACTAGGACCACAACTTCGGAGCAGGGCAA
 -2fr D··V··H··D··V··M··A··N··S··T··R··H··H··R··V··D··A··R··Q··E··I··I··R··T··N··F··G··R··G··T··

+3fr ·T··K··I··G··L··I··A··T··A··A··G··Y··A··I··Y··C··T··V··G··V··A··F··Q··L··L··L··V··S··Y··H·

     541 TCACCAAAATAGGCCTTATCGCAACCGCTGCAGGGTATGCGATATACTGCACTGTTGGGGTTGCTTTTCAACTGCTTCTTGTCTCGTATC
     541 AGTGGTTTTATCCGGAATAGCGTTGGCGACGTCCCATACGCTATATGACGTGACAACCCCAACGAAAAGTTGACGAAGAACAGAGCATAG
 -2fr E··G··F··Y··A··K··D··C··G··S··C··P··I··R··Y··V··A··S··N··P··N··S··K··L··Q··K··K··D··R··I··

+2fr  M··C··W··R··L··S··L··F··P··K··Y··M··L··I··A··W··E··T··L··H··G··G··S·
 +3fr ·I··V··Y··L··F··P··G··C··A··G··D··F··H··F··F··Q··S··I··C··

     631 ATATCGTGTATCTTTTCCCCGGATGTGCTGGCGACTTTCACTTTTTCCAAAGTATATGCTGATCGCCTGGGAAACGTTACATGGAGGCAG
     631 TATAGCACATAGAAAAGGGGCCTACACGACCGCTGAAAGTGAAAAAGGTTTCATATACGACTAGCGGACCCTTTGCAATGTACCTCCGTC
 -2fr ·M

+2fr  ·M··R··K··S··E··E··V··T··G··V··D··L··A··S··M··F··S··G··F··F··L··G··F··S··R··R··P··F··G··Y·
     721 TATGAGGAAGTCAGAGGAAGTCACTGGGGTTGATCTTGCAAGTATGTTCTCCGGCTTCTTCCTGGGGTTTAGCAGGAGACCTTTTGGGTA
     721 ATACTCCTTCAGTCTCCTTCAGTGACCCCAACTAGAACGTTCATACAAGAGGCCGAAGAAGGACCCCAAATCGTCCTCTGGAAAACCCAT

+2fr  ·L··C··C··I··K··E··L··I··T··Y··E··E··S··S··A··G··R··H··D··G··Y··D··P··N··C··G··S··S··E··G·
 +3fr M··K··K··A··V··Q··V··A··M··T··A··M··T··Q··T··V··A··A··L··K··G

811 CTTATGTTGCATAAAAGAATTAATTACATATGAAGAAAGCAGTGCAGGTCGCCATGACGGCTATGACCCAAACTGTGGCAGCTCTGAAGG
     811 GAATACAACGTATTTTCTTAATTAATGTATACTTCTTTCGTCACGTCCAGCGGTACTGCCGATACTGGGTTTGACACCGTCGAGACTTCC

+2fr  ·K··E··A··R··S··L··Q··T··E··N··E··Q··H··S··P··P··

+3fr ·R··K··L··E··A··S··K··P··K··T··S··S··T··P··L··P··K··S··S··D··G··R··L··A··S··G··E··A··S··R·

901 GAAGGAAGCTAGAAGCCTCCAAACCGAAAACGAGCAGCACTCCCCTCCCTAAATCCTCAGATGGCAGGCTAGCTTCGGGAGAAGCATCAC
 901 CTTCCTTCGATCTTCGGAGGTTTGGCTTTTGCTCGTCGTGAGGGGAGGGATTTAGGAGTCTACCGTCCGATCGAAGCCCTCTTCGTAGTG

+3fr ·E··T··P··A··S··L··S··G··Q··A··E··T··V··Q··Q··K··P··P··L··Q··A··E··S··V··Q··P··K··S··G··P·

991 GTGAAACCCCAGCTAGTCTGTCAGGACAGGCAGAAACTGTGCAGCAGAAGCCCCCTCTGCAGGCCGAGTCTGTGCAGCCAAAGTCTGGTC
     991 CACTTTGGGGTCGATCAGACAGTCCTGTCCGTCTTTGACACGTCGTCTTCGGGGGAGACGTCCGGCTCAGACACGTCGGTTTCAGACCAG

+3fr ·L··T··R··S··G··T··S··N··Q··G··S··T··P··F··G··R··P··L··I··R··V··S··H··L··E··E··V··V··Q··S·

    1081 CCTTGACCCGAAGTGGCACCTCCAACCAAGGCTCTACACCTTTCGGTAGACCCTTAATCAGAGTGTCTCACTTAGAGGAAGTTGTACAAT
    1081 GGAACTGGGCTTCACCGTGGAGGTTGGTTCCGAGATGTGGAAAGCCATCTGGGAATTAGTCTCACAGAGTGAATCTCCTTCAACATGTTA

+2fr M··A··H··N·

+3fr ·S··I··N··E··D··L··Y··L··S··D··A··T··S··T··G··A··S··E··A··E··A··S··D··

    1171 CCTCAATCAACGAGGATCTATATTTATCTGATGCCACCAGCACTGGGGCATCTGAAGCAGAAGCAAGTGACTAACCATCATGGCACACAA
    1171 GGAGTTAGTTGCTCCTAGATATAAATAGACTACGGTGGTCGTGACCCCGTAGACTTCGTCTTCGTTCACTGATTGGTAGTACCGTGTGTT

+2fr ·L··S··I··L··Q··W··N··I··C··S··Y··K··S··K··N··S··F··L··Q··S··V··V··G··A··R··S··I··D··V··M·

    1261 TCTAAGCATTCTGCAGTGGAATATCTGTAGCTATAAAAGCAAGAACTCCTTTCTCCAGTCAGTTGTGGGAGCAAGAAGCATTGACGTCAT
    1261 AGATTCGTAAGACGTCACCTTATAGACATCGATATTTTCGTTCTTGAGGAAAGAGGTCAGTCAACACCCTCGTTCTTCGTAACTGCAGTA

+2fr ·L··R··E··T··L··T··M··G··S··V··C··F··S··G··Y··H··A··F··T··T··P··N··L··D··G··A··R··G··L··M·
    1351 GCTCCGGGAGACACTAACTATGGGATCCGTGTGCTTCTCAGGATATCATGCCTTCACCACTCCAAACCTGGATGGTGCTAGAGGCCTGAT
    1351 CGAGGCCCTCTGTGATTGATACCCTAGGCACACGAAGAGTCCTATAGTACGGAAGTGGTGAGGTTTGGACCTACCACGATCTCCGGACTA

+2fr ·T··L··V··K··E··A··I··P··C··S··L··I··A··N··P··P··H··C··G··D··V··E··S··L··A··V··E··I··Q··L·
    1441 GACCCTGGTAAAAGAAGCTATCCCGTGCTCCTTGATAGCCAACCCGCCGCACTGTGGAGATGTTGAATCTCTTGCTGTTGAGATTCAGCT
    1441 CTGGGACCATTTTCTTCGATAGGGCACGAGGAACTATCGGTTGGGCGGCGTGACACCTCTACAACTTAGAGAACGACAACTCTAAGTCGA

+2fr ·P··G··G··P··I··K··I··Y··N··I··Y··S··K··P··L··C··E··S··L··D··L··N··Q··V··C··A··I··A··A··Q·
    1531 ACCTGGGGGCCCTATAAAAATATATAATATTTATAGCAAACCACTGTGTGAGAGCTTAGATCTGAACCAGGTCTGTGCTATTGCAGCACA
    1531 TGGACCCCCGGGATATTTTTATATATTATAAATATCGTTTGGTGACACACTCTCGAATCTAGACTTGGTCCAGACACGATAACGTCGTGT

+2fr ·D··R··V··I··I··G··V··D··F··N··A··H··I··A··M··L··N··S··R··K··R··P··N··A··A··G··I··H··I··A·
    1621 AGACCGAGTGATCATAGGGGTAGACTTCAATGCACACATAGCCATGCTGAATTCCCGCAAAAGGCCGAACGCGGCAGGCATTCACATAGC
    1621 TCTGGCTCACTAGTATCCCCATCTGAAGTTACGTGTGTATCGGTACGACTTAAGGGCGTTTTCCGGCTTGCGCCGTCCGTAAGTGTATCG

+2fr ·E··V··L··E··T··F··P··E··I··A··L··L··N··T··K··E··P··T··H··V··K··G··G··V··L··D··L··T··F··A·

    1711 AGAAGTGCTTGAGACATTCCCTGAGATCGCTCTTCTCAACACCAAAGAACCAACGCATGTGAAGGGAGGGGTCCTAGACCTCACCTTTGC
    1711 TCTTCACGAACTCTGTAAGGGACTCTAGCGAGAAGAGTTGTGGTTTCTTGGTTGCGTACACTTCCCTCCCCAGGATCTGGAGTGGAAACG

+2fr ·T··A··T··M··V··E··R··I··R··W··C··V··D··D··T··V··T··S··D··H··Y··G··I··V··T··T··L··M··D··A·
    1801 CACTGCAACAATGGTGGAGAGAATTCGGTGGTGTGTCGATGATACGGTTACTAGTGATCACTACGGCATAGTCACCACACTAATGGATGC
    1801 GTGACGTTGTTACCACCTCTCTTAAGCCACCACACAGCTACTATGCCAATGATCACTAGTGATGCCGTATCAGTGGTGTGATTACCTACG

+2fr ·G··P··A··Q··R··P··H··H··I··P··K··W··K··T··D··K··A··N··W··F··A··F··Q··E··G··L··A··R··C··L·
    1891 AGGTCCAGCCCAAAGACCACACCACATTCCTAAATGGAAAACAGACAAGGCCAACTGGTTTGCCTTCCAAGAAGGCTTGGCTCGATGTCT
    1891 TCCAGGTCGGGTTTCTGGTGTGGTGTAAGGATTTACCTTTTGTCTGTTCCGGTTGACCAAACGGAAGGTTCTTCCGAACCGAGCTACAGA

+2fr ·K··D··N··E··P··N··N··N··N··E··N··V··D··V··L··E··A··R··L··I··Q··A··I··N··Q··A··A··S··Q··T·
    1981 GAAAGACAATGAACCCAATAACAATAATGAAAACGTGGATGTGCTAGAGGCAAGGCTAATCCAGGCCATAAACCAGGCAGCCTCACAAAC
    1981 CTTTCTGTTACTTGGGTTATTGTTATTACTTTTGCACCTACACGATCTCCGTTCCGATTAGGTCCGGTATTTGGTCCGTCGGAGTGTTTG

+2fr ·I··P··K··T··R··P··W··S··R··T··H··K··D··A··W··Y··Y··N··D··E··I··K··E··V··N··H··R··V··N··M·
    2071 CATTCCCAAAACTCGTCCATGGTCCAGAACTCACAAAGACGCCTGGTACTATAATGACGAGATCAAAGAGGTCAACCACCGGGTTAATAT
    2071 GTAAGGGTTTTGAGCAGGTACCAGGTCTTGAGTGTTTCTGCGGACCATGATATTACTGCTCTAGTTTCTCCAGTTGGTGGCCCAATTATA

+2fr ·C··R··K··N··F··R··R··Q··R··S··P··D··N··L··A··L··L··R··E··A··V··V··D··A··K··E··T··T··N··R·
    2161 GTGTAGAAAAAACTTCCGACGGCAAAGATCTCCCGACAATCTGGCCCTGCTGAGGGAAGCTGTTGTGGATGCCAAGGAAACTACCAACAG
    2161 CACATCTTTTTTGAAGGCTGCCGTTTCTAGAGGGCTGTTAGACCGGGACGACTCCCTTCGACAACACCTACGGTTCCTTTGATGGTTGTC

+2fr ·V··R··Q··E··K··W··L··E··W··C··Q··S··F··G··Y··Q··T··S··L··T··E··L··W··K··R··V··R··Q··A··T·
    2251 AGTAAGGCAGGAAAAATGGCTGGAATGGTGCCAGTCTTTTGGGTACCAGACCAGCCTTACAGAGTTGTGGAAACGGGTCAGGCAAGCGAC
    2251 TCATTCCGTCCTTTTTACCGACCTTACCACGGTCAGAAAACCCATGGTCTGGTCGGAATGTCTCAACACCTTTGCCCAGTCCGTTCGCTG

+2fr ·S··R··Q··A··P··K··C··T··H··H··D··P··Q··S··E··A··N··R··L··V··L··E··F··S··A··R··T··S··T··N·
    2341 AAGCCGCCAAGCCCCGAAATGCACTCATCATGACCCACAATCAGAGGCAAATAGATTGGTGCTTGAGTTCTCTGCCAGAACCAGCACCAA
    2341 TTCGGCGGTTCGGGGCTTTACGTGAGTAGTACTGGGTGTTAGTCTCCGTTTATCTAACCACGAACTCAAGAGACGGTCTTGGTCGTGGTT

+2fr ·N··L··P··P··M··M··R··E··K··Q··Q··N··L··N··P··E··R··L··A··L··I··R··D··K··A··L··E··A··D··E·
    2431 CAATCTGCCTCCAATGATGAGAGAAAAACAACAAAACTTAAATCCAGAAAGACTTGCTCTCATAAGAGACAAGGCACTCGAAGCTGATGA
    2431 GTTAGACGGAGGTTACTACTCTCTTTTTGTTGTTTTGAATTTAGGTCTTTCTGAACGAGAGTATTCTCTGTTCCGTGAGCTTCGACTACT

+2fr ·A··D··A··L··F··S··L··R··E··L··R··K··S··Y··E··S··S··S··G··S··A··P··G··S··D··G··I··S··H··P·
    2521 AGCAGATGCCTTGTTCTCTCTTAGGGAACTAAGAAAATCATACGAATCCAGTTCTGGGTCAGCACCGGGATCTGATGGGATCTCTCACCC
    2521 TCGTCTACGGAACAAGAGAGAATCCCTTGATTCTTTTAGTATGCTTAGGTCAAGACCCAGTCGTGGCCCTAGACTACCCTAGAGAGTGGG

+2fr ·I··I··S··H··L··G··L··A··G··E··L··A··F··L··Q··V··I··Y··K··S··W··Q··T··A··T··V··P··Q··S··W·
    2611 CATCATTTCGCATCTAGGCCTTGCAGGAGAACTTGCATTCTTGCAGGTTATCTACAAATCCTGGCAAACAGCCACGGTGCCCCAGAGCTG
    2611 GTAGTAAAGCGTAGATCCGGAACGTCCTCTTGAACGTAAGAACGTCCAATAGATGTTTAGGACCGTTTGTCGGTGCCACGGGGTCTCGAC

+2fr ·K··Q··A··T··I··V··P··I··P··K··P··K··E··P··G··K··Y··R··P··I··S··L··L··S··C··L··G··K··T··A·
    2701 GAAACAAGCCACAATAGTTCCCATCCCAAAGCCAAAGGAGCCTGGCAAGTACCGCCCCATCTCTCTTCTCAGCTGCCTGGGTAAAACAGC
    2701 CTTTGTTCGGTGTTATCAAGGGTAGGGTTTCGGTTTCCTCGGACCGTTCATGGCGGGGTAGAGAGAAGAGTCGACGGACCCATTTTGTCG

+2fr ·E··K··M··V··L··N··R··L··R··W··K··T··G··P··P··H··E··H··L··H··G··F··T··R··G··K··S··T··A··H·
    2791 TGAGAAGATGGTACTCAACAGGCTCCGATGGAAAACGGGTCCCCCCCATGAACACCTGCACGGGTTCACAAGGGGTAAGAGCACTGCTCA
    2791 ACTCTTCTACCATGAGTTGTCCGAGGCTACCTTTTGCCCAGGGGGGGTACTTGTGGACGTGCCCAAGTGTTCCCCATTCTCGTGACGAGT

+2fr ·S··I··S··T··L··L··S··T··I··C··T··S··P··A··V··V··V··F··L··D··L··E··K··A··F··E··L··A··S··P·
    2881 TAGCATTTCCACACTCTTAAGCACAATCTGTACCTCGCCCGCCGTGGTTGTCTTCCTTGACCTGGAGAAGGCTTTTGAGTTGGCAAGTCC
    2881 ATCGTAAAGGTGTGAGAATTCGTGTTAGACATGGAGCGGGCGGCACCAACAGAAGGAACTGGACCTCTTCCGAAAACTCAACCGTTCAGG

+2fr ·L··A··I··Q··E··T··L··I··H··K··G··V··K··G··R··L··L··A··W··I··A··D··Y··F··K··N··R··S··A··N·
    2971 ACTAGCCATTCAAGAGACCCTAATCCACAAAGGAGTCAAAGGCAGACTCTTGGCCTGGATAGCTGACTATTTTAAAAATAGATCAGCAAA
    2971 TGATCGGTAAGTTCTCTGGGATTAGGTGTTTCCTCAGTTTCCGTCTGAGAACCGGACCTATCGACTGATAAAATTTTTATCTAGTCGTTT

+2fr ·V··R··F··Q··G··H··L··S··Q··H··M··P··L··E··N··G··T··P··Q··G··G··V··L··S··P··A··L··F··N··T·

    3061 TGTCAGATTTCAAGGCCACCTCTCACAGCACATGCCACTTGAAAATGGAACTCCTCAGGGAGGGGTTCTTAGTCCAGCCCTGTTTAATAC
    3061 ACAGTCTAAAGTTCCGGTGGAGAGTGTCGTGTACGGTGAACTTTTACCTTGAGGAGTCCCTCCCCAAGAATCAGGTCGGGACAAATTATG

+2fr ·L··M··S··N··I··L··D··I··H··L··P··E··G··C··K··I··I··S··Y··A··D··D··L··A··I··I··A··S··G··N·

    3151 CCTCATGTCCAACATACTCGACATTCACCTGCCAGAGGGATGCAAGATCATCTCTTATGCAGATGACTTGGCAATCATAGCCTCTGGCAA
    3151 GGAGTACAGGTTGTATGAGCTGTAAGTGGACGGTCTCCCTACGTTCTAGTAGAGAATACGTCTACTGAACCGTTAGTATCGGAGACCGTT

+2fr ·H··C··L··T··R··A··Q··R··C··L··N··L··V··S··E··E··C··C··R··T··G··L··K··I··S··A··A··K··S··K·
    3241 TCACTGCCTTACTAGAGCTCAGCGTTGTCTGAACCTGGTGTCCGAAGAGTGTTGTAGGACGGGTCTAAAAATATCAGCAGCAAAATCCAA
    3241 AGTGACGGAATGATCTCGAGTCGCAACAGACTTGGACCACAGGCTTCTCACAACATCCTGCCCAGATTTTTATAGTCGTCGTTTTAGGTT

+2fr ·A··M··A··L··R··T··N··V··R··N··K··K··L··T··I··Q··G··M··D··L··E··

3331 AGCAATGGCTTTGAGAACCAATGTCAGAAACAAAAAACTCACTATACAGGGTATGGATCTGGAATGAGTGAAGGATTATCTATACCTTGG
    3331 TCGTTACCGAAACTCTTGGTTACAGTCTTTGTTTTTTGAGTGATATGTCCCATACCTAGACCTTACTCACTTCCTAATAGATATGGAACC

+2fr M··E··I··Q··Y··L··L··D··R··T··E··E··R··P··S··V··M··K··V··L·
    3421 TGTATGGATAGGACACACACTCACTTTCAAAATGGAGATCCAATACCTGCTTGACAGAACCGAGGAAAGACCGTCAGTCATGAAAGTCTT
    3421 ACATACCTATCCTGTGTGTGAGTGAAAGTTTTACCTCTAGGTTATGGACGAACTGTCTTGGCTCCTTTCTGGCAGTCAGTACTTTCAGAA

+2fr ·T··G··I··H··I··G··A··G··H··K··V··L··R··S··F··Y··V··H··A··V··R··P··I··I··D··Y··A··S··V··A·
    3511 GACAGGGATACACATAGGAGCAGGACACAAAGTACTAAGATCATTCTATGTACATGCTGTCCGTCCTATTATTGACTATGCATCTGTCGC
    3511 CTGTCCCTATGTGTATCCTCGTCCTGTGTTTCATGATTCTAGTAAGATACATGTACGACAGGCAGGATAATAACTGATACGTAGACAGCG

+2fr ·L··I··A··S··S··T··T··L··K··E··K··L··E··I··I··Q··N··E··G··A··R··I··I··L··G··A··P··K··W··T·
    3601 CCTCATTGCTTCCAGCACAACATTAAAAGAAAAACTGGAAATAATACAAAACGAAGGAGCCAGAATAATTCTAGGTGCACCTAAGTGGAC
    3601 GGAGTAACGAAGGTCGTGTTGTAATTTTCTTTTTGACCTTTATTATGTTTTGCTTCCTCGGTCTTATTAAGATCCACGTGGATTCACCTG

+2fr ·K··V··I··N··L··L··M··E··T··D··L··P··S··M··D··T··R··I··D··L··M··V··A··Q··F··I··S··K··V··L·
    3691 AAAGGTCATCAACCTCCTCATGGAAACTGATTTACCGTCCATGGACACTAGAATTGATCTAATGGTAGCACAATTCATTTCCAAGGTCCT
    3691 TTTCCAGTAGTTGGAGGAGTACCTTTGACTAAATGGCAGGTACCTGTGATCTTAACTAGATTACCATCGTGTTAAGTAAAGGTTCCAGGA

+2fr ·Q··A··P··T··N··S··Y··L··R··Q··R··V··L··R··R··L··Q··Q··D··Y··Q··L··F··A··D··N··S··W··L··T·
    3781 GCAGGCACCCACAAACTCCTATCTAAGACAAAGAGTTCTCAGACGCCTACAACAAGATTACCAGTTGTTTGCGGACAATTCCTGGCTCAC
    3781 CGTCCGTGGGTGTTTGAGGATAGATTCTGTTTCTCAAGAGTCTGCGGATGTTGTTCTAATGGTCAACAAACGCCTGTTAAGGACCGAGTG

+2fr ·H··T··A··R··V··L··I··R··F··Q··L··K··L··S··L··L··A··K··G··M··D··S··P··H··P··D··Y··K··E··P·
    3871 ACATACAGCCAGAGTTTTAATACGCTTTCAGCTAAAACTTTCATTGCTTGCCAAGGGTATGGACTCCCCTCACCCTGATTACAAAGAACC
    3871 TGTATGTCGGTCTCAAAATTATGCGAAAGTCGATTTTGAAAGTAACGAACGGTTCCCATACCTGAGGGGAGTGGGACTAATGTTTCTTGG

+2fr ·H··R··E··R··G··F··F··V··I··R··V··S··Q··G··S··R··F··R··I··L··S··S··K··S··H··N··E··K··R··S·
 +3fr M··R··K··D··Q

    3961 CCACCGTGAGCGGGGGTTCTTTGTAATCAGGGTGAGTCAAGGTAGTCGTTTTAGAATTCTCAGTTCTAAATCTCACAATGAGAAAAGATC
    3961 GGTGGCACTCGCCCCCAAGAAACATTAGTCCCACTCAGTTCCATCAGCAAAATCTTAAGAGTCAAGATTTAGAGTGTTACTCTTTTCTAG

+2fr ·I··F··H··A··Y··P··K··G··T··S··T··K··G··H··

+3fr ·Y··S··M··P··I··L··K··A··Q··A··Q··R··V··I··E··Q··I··T··P··P··G··S··I··T··Y··Y··T··D··G··S·

    4051 AATATTCCATGCCTATCCTAAAGGCACAAGCACAAAGGGTCATTGAACAAATAACTCCGCCGGGTAGTATAACTTACTACACGGACGGAT
    4051 TTATAAGGTACGGATAGGATTTCCGTGTTCGTGTTTCCCAGTAACTTGTTTATTGAGGCGGCCCATCATATTGAATGATGTGCCTGCCTA

+3fr ·V··D··P··I··N··H··T··A··G··A··G··F··A··T··K··D··T··T··A··S··I··R··V··T··D··N··A··S··T··L·

    4141 CCGTGGATCCAATAAACCATACTGCAGGCGCCGGCTTTGCAACAAAGGATACCACAGCATCCATTAGGGTCACTGACAATGCCTCAACGC
    4141 GGCACCTAGGTTATTTGGTATGACGTCCGCGGCCGAAACGTTGTTTCCTATGGTGTCGTAGGTAATCCCAGTGACTGTTACGGAGTTGCG

+3fr ·Q··A··E··T··V··A··I··M··E··A··L··T··H··A··S··L··R··G··G··H··V··V··I··H··T··D··S··R··A··T·

    4231 TTCAGGCTGAAACGGTTGCGATCATGGAAGCATTGACACACGCGTCCCTGAGGGGAGGGCACGTTGTCATTCATACAGATTCAAGAGCAA
    4231 AAGTCCGACTTTGCCAACGCTAGTACCTTCGTAACTGTGTGCGCAGGGACTCCCCTCCCGTGCAACAGTAAGTATGTCTAAGTTCTCGTT

+3fr ·I··D··S··L··Q··H··S··M··P··P··D··N··I··Y··L··L··T··T··V··L··Y··I··A··Q··R··I··L··S··Q··G·

    4321 CTATTGACAGTTTACAGCATAGCATGCCCCCAGATAACATCTACCTCTTGACAACAGTACTATACATAGCCCAAAGAATCCTTAGTCAAG
    4321 GATAACTGTCAAATGTCGTATCGTACGGGGGTCTATTGTAGATGGAGAACTGTTGTCATGATATGTATCGGGTTTCTTAGGAATCAGTTC

+3fr ·R··R··I··I··I··N··W··V··P··S··H··I··G··I··Q··G··N··E··L··A··D··K··L··A··E··K··G··R··G··M·
    4411 GTAGAAGAATCATCATAAACTGGGTCCCAAGCCACATAGGCATACAAGGGAACGAGCTTGCTGATAAACTAGCCGAAAAGGGCAGGGGTA
    4411 CATCTTCTTAGTAGTATTTGACCCAGGGTTCGGTGTATCCGTATGTTCCCTTGCTCGAACGACTATTTGATCGGCTTTTCCCGTCCCCAT

+3fr ·T··P··S··S··M··I··V··K··P··S··R··R··G··L··S··Q··G··T··K··A··A··A··C··A··V··L··R··G··A··H·
    4501 TGACCCCATCCTCCATGATCGTTAAACCCAGCCGAAGGGGACTGAGCCAAGGTACCAAAGCTGCAGCGTGTGCGGTCCTCCGGGGAGCAC
    4501 ACTGGGGTAGGAGGTACTAGCAATTTGGGTCGGCTTCCCCTGACTCGGTTCCATGGTTTCGACGTCGCACACGCCAGGAGGCCCCTCGTG

+3fr ·R··E··N··I··T··K··S··L··A··A··K··W··Y··S··D··A··S··G··Y··E··P··L··A··L··P··P··N··T··K··R·

    4591 ATAGAGAAAACATCACAAAATCGCTCGCTGCCAAGTGGTACTCAGATGCTTCAGGCTATGAGCCACTGGCCCTTCCTCCAAATACAAAAA
    4591 TATCTCTTTTGTAGTGTTTTAGCGAGCGACGGTTCACCATGAGTCTACGAAGTCCGATACTCGGTGACCGGGAAGGAGGTTTATGTTTTT

+3fr ·G··T··E··V··I··L··F··R··L··R··L··G··Y··Q··C··A··W··Q··I··I··D··S··E··S··G··R··S··C··K··H·

    4681 GAGGTACCGAAGTCATACTATTCAGACTAAGACTAGGTTACCAATGCGCTTGGCAAATTATTGACAGTGAGTCTGGGAGGTCATGTAAAC
    4681 CTCCATGGCTTCAGTATGATAAGTCTGATTCTGATCCAATGGTTACGCGAACCGTTTAATAACTGTCACTCAGACCCTCCAGTACATTTG

+3fr ·C··G··E··P··N··A··T··L··L··H··Y··L··Q··N··C··V··H··T··Q··F··L··R··Q··G··P··P··T··T··A··A·

    4771 ACTGCGGCGAGCCTAACGCCACCCTGTTACATTACTTGCAGAATTGTGTACACACACAATTTCTGAGACAGGGACCACCCACCACAGCCG
    4771 TGACGCCGCTCGGATTGCGGTGGGACAATGTAATGAACGTCTTAACACATGTGTGTGTTAAAGACTCTGTCCCTGGTGGGTGGTGTCGGC

+3fr ·G··L··V··K··R··V··G··N··M··L··S··P··W··Q··L··D··R··L··L··A··I··Q··P··P··R··

    4861 CCGGGCTGGTAAAAAGGGTGGGCAACATGCTTTCACCATGGCAGCTGGATCGCTTGCTTGCAATCCAGCCACCGCGGTAAGCATGCAGTT
    4861 GGCCCGACCATTTTTCCCACCCGTTGTACGAAAGTGGTACCGTCGACCTAGCGAACGAACGTTAGGTCGGTGGCGCCATTCGTACGTCAA

    4951 CAAAGAAAACATTTGAGTTAACTAGAAATATTTAACACAGGCCGGGCCACCCCAGAGAAGAGGCCCGGGCGAAGCATGACTTCGCAAATC
    4951 GTTTCTTTTGTAAACTCAATTGATCTTTATAAATTGTGTCCGGCCCGGTGGGGTCTCTTCTCCGGGCCCGCTTCGTACTGAAGCGTTTAG

    5041 TAACTGAACTGA
 5041 ATTGACTTGACT
