# Supplementary material for: Discovery and partial characterization of a non-LTR retrotransposon that may be associated with abdominal segment deformity disease (ASDD) in the whiteleg shrimp Penaeus (Litopenaeus) vannamei
Source: BMC Vet Res. 2013 Sep 30;9:189. doi: 10.1186/1746-6148-9-189 (PMC3849965; doi:10.1186/1746-6148-9-189)
Supplement: Additional file 3 — Alignment of ASDE and a PCR amplicon sequence. Clustral alignment of the ASDE sequence (5052 bp) and a clone of the 4091 bp PCR amplicon obtained using primers designed to amplify the whole 5052 sequence from a total DNA template extracted from ASDD shrimp. The location of the reverse transcriptase target for RT-PCR, PCR and the hybridization probe is indicated in grey outline and the primer sequences are underlined. [file 1746-6148-9-189-S3.doc]

ASDE CGGCCTGTGCAGACTAATGTCGACTCGATCAACGTGTATCTACTAGTGCTGACGCGACAG 60

4K CGGCCTGTGCAGACTAATGTCGACTCGATCAACGTGTGTCAGCGAGTGCTGACGCAACAG 60

************************************* ** * *********** ****

ASDE AGCTTAGTCCTTACCCACCGCCGTGCCCAGCCACAGCAGTAACCTCCGAGCGACAATTGC 120

4K AGCTTAGTCCTTCCCCACCGTGGTGCCCAACTACAGCAGTAACCTCCGGGCGACAATTGC 120

************ ******* ******* * **************** ***********

ASDE AACTTCTCGCGCCTGGGCGGGGAGCGAACCGCCGACCCCTCGGATGAGAGGACGGCACGT 180

4K AACTTCTCGCGCCTGGGCGGGGCGCGAACCGCCGACCCCTCGGATG---------CGCAA 171

********************** *********************** * *

ASDE TACCACTGTACTAGCCTGGAGGCTACGTAGCATATATATACTACATGGGAGCTGGTAAAT 240

4K TCCCAC-GCACGAGCT-------------GCATCTAAACAAAACA-----------AAAA 206

* **** * ** *** **** ** * * *** ***

ASDE CTCCTGACGACTGTGTCCTCGCACTCGTTACGCGCATAATTGCAGCTGCGACCTTGGATA 300

4K CTTCTA-----------CTCGCACTAACAACAAACACTA--ACAAACACAACTCCGGCTT 253

** ** ******** ** ** * ** * ** ** *

ASDE CTTTAAACCTGCCTGATGCGGTGTTCATATTCTTTTCTGTCGCGATGTATGCAGCTTCCA 360

4K GTTCTGA--TGG--AATAAAGCACTCAGACGGTTCCCTGTAGTGG-GT----------CG 298

** * ** ** * *** * ** **** * * ** *

ASDE TGACCTTCCTTTTATGTTTACTCACTCTTTCATGGATTAATTTGGCTTCCTTCCAG---T 417

4K TGACCGATCTATTACATGTCTGAACTCTGCGA-GGAAGCCTCTTGCTTCCTCCTTAACAT 357

***** ** *** * * ***** * *** * * ******* * *

ASDE TCGGTAGATGTCCAGCTTCATCTACATGATCTACCATGGCATTGGAAGTCCTGTGGT-GA 476

4K CCTGTGG--GGCGAG-TTGGCCTGTA-GACAGACCA-------ACAAACTCCGTACTTGA 406

* ** * * * ** ** ** * ** **** ** * ** * **

ASDE CGGACGTCGGCTCGCTGTTCGATGATCCTGGTGTTGAAGCCTCGTCCCGTTTCACCAAAA 536

4K CTCACC---ACTGGCTG---GATGGACTCACCCTCCATCCTTCGTTCCGGCTGG--AGAT 458

* ** ** **** **** * * * * **** *** * * *

ASDE TAGGCCTTA-TCGCAACCGCTGCAG--GGTATGCGATAT--ACTGCACTGTTGGGGTTGC 591

4K CAGGTTTAGGCCGTGATCTCTTTCTTTGGCATGGGACAGGAAATGCAGTTTTCGGGTT-- 516

*** * ** * * ** ** *** ** * * **** * ** *****

ASDE TTTTCAACTGCTTCTTGTCTCGTATCATATCGTG--TATCTTTTCCCCGGATGTGCTGGC 649

4K --------------TTGC--CGCATCACTTTGAAGCCACCTTCTACTC--AT-TCCTTTC 557

*** ** **** * * * *** * * * ** * ** *

ASDE GACTTTCACTTTTTCCAAAGTATATGCTGATCGCCTGGGAAACGTTACATGGAGGCAGTA 709

4K AAATTTCCCCTTCCCC------TAT-CCACTCGTTTTGTTCA-GTTTTTT-----CAGTG 604

* **** * ** ** *** * *** * * * *** * ****

ASDE TGAGGAAGTCAGAGGAAGTCACTGGGGTTGATCTTGCAAGTATGTTCTCCGGCTTCTTCC 769

4K T-------TCA------GTTTTTCGTGTTC-TCTTTC---TGTCTGCTTAG---TTTTGT 644

* *** ** * * *** **** * * * * ** * * **

ASDE TGGGGTTTAGCAGGAGACCTTTTGGGTACTTATGTTGCATAAAAGAATTAATTACATATG 829

4K TGTTGTTTGGT--------TTTTG-----TTGTCCTGTACCTTT---TTAATCTCAC--T 686

** **** * ***** ** * ** * ***** **

ASDE AAGAAAGCAGTGCAGGTCGCCATGACGGCTATGACCCAAACTGTGGCAGCTCTGAAGGGA 889

4K AATAAAGTAATCT---CCCCCA----GGTACTGCCCTCTCTTATCCCCGTTTTTAAATAC 739

** **** * * * *** ** ** ** * * * * * * **

ASDE AGGAAGCTAGAAGCCTCCAAACCGAAAACGAGCAGCACTCCCCTCCCTAAATCCTCAG-- 947

4K CTTCACCCCG---TCTCCTGAC-GCTTGTGACCACCACCTCACCCCTCA--CCCTCACTC 793

* * * **** ** * ** ** *** * * ** * *****

ASDE ATGGCAGGCTAGCTTCGGGAGAAGCATCACGTGAAACCCCAGC-TAGTC--TGTCAGGAC 1004

4K ATACCA---TATCCCTGC------CATGGCGTTGACCCTCATCCTTATCCTTACCCTGAC 844

** ** ** * * *** *** * ** ** * * ** * * ***

ASDE AGGCAGAAACTGTGCAGCAGAAGCCCCCTCTGCAGGCCGAGTCTGTGCAGCCAAAGTCTG 1064

4K ATCC---CACTG----GTTTCGGTTTCCGACACATGGCG---CT-TGCTGCTAGTA-CTC 892

* * **** * * ** ** * ** ** *** ** * **

ASDE GTCCCTTGACCCGAAGTGGCACCTCCAAC-CAAGGCTCTACACCTTTCGGTAGACCCTTA 1123

4K GTGCCAGGACCACAAAT----CCTTTAGCGTAAGGGTTTGTGCGAGCCGA-GGA-----A 942

** ** **** ** * *** * * **** * * * ** ** *

ASDE ATCAGAGTGTCTCACTTAGAGGAAGTTGTACAATCCTCAATCAACGAGGATCTATATTTA 1183

4K A--GAAGCGTCACGGCCAGTGGAGTTAACCCAATTGT-----GGTGAGG--------CCG 987

* ** *** * ** *** * **** * ****

ASDE TCTGATGCCAC-CAGCACTGGGGCATCTGAAGCAGAAGCAAGTGACTAACCATCATGGCA 1242

4K CCCGA-GCCGCGCCCCTCCGGAAGACCTGCGGCCGAGG-GAGAGA--AATC-TCCT---- 1038

* ** *** * * * * ** * *** ** ** * ** ** ** * ** *

ASDE CACAATCTAAGC---ATTCTGCAGTGGAATATCTGTAGCTATAAAAGCAAGAACTCCTTT 1299

4K CTCGACCCGGGCGGGACTAGCCAGC---CTCTGTGTGGCCTCA----CTCGAGCTTCATC 1091

* * * * ** * * *** * * *** ** * * ** ** * *

ASDE CTCCAGTCAGTTGTGGGAGCAAGAAGCATTGACGTCATGCTCCGGGAGACACTAACTATG 1359

4K CTGCT-TCAGCGGTGATGGTTAG------------------CCGAGGGACA---GCTCTG 1129

** * **** *** * ** *** * **** ** **

ASDE GGATCCGTGTGCTTCTCAGGATATCATGCCTTC-ACCACTCCAAACCTGGATGGTGCTAG 1418

4K G-AGCCACG-GCCTCTCG----ACCGTGGCTCCGAGCATACCG----CAGATGGCGCCAA 1179

* * ** * ** **** * * ** ** * * ** ** ***** ** *

ASDE AGGCCTGATGACCCTGGTAAAAGAAGCTATCCCGTGCTCCTTGATAGCCAACCCGCCGCA 1478

4K AAACCTGCTAACTT------------CTCTCCCCT-CTCCCT--CAGTTAGTC------- 1217

* **** * ** ** **** * **** * ** * *

ASDE CTGTGGAGATGTTGAATCTCTTGCTGT--TGAGATTCAGCTACCTGGGGGCCCTATAAAA 1536

4K -----GAGAGGCTCCGTGT-TCGCCATAGTGAGGCTCCACGCCCCATGGG------AAAA 1265

**** * * * * * ** * **** ** * ** *** ****

ASDE ATATATAATATTTATAGCAAACCACTGTGTGAGAGCTTAGATCTGAACCAGGTC-TGTGC 1595

4K ATGTGG------TGAGGCGAGCCGC---------GCTCA----------AGGTCGTGGGG 1300

** * * ** * ** * *** * ***** ** *

ASDE TATT----GCAGCACAAGACCGAGTGATCATAGGGGTAGACTTCAATGCACACATAGCCA 1651

4K TATTCCCCGCGCCACGAGTC---GCGGCCTTG--------CCCCACCGCAC--------- 1340

**** ** *** ** * * * * * * ** ****

ASDE TGCTGAATTCCCGCAAAA---GGCCGAACGCGGCAGGCATTCACATA-GCAGA-AGTGCT 1706

4K ----GA--TCTCGCGAGGTACGGCCG--CGCGGTGAACACGCGAGTCTGCCGATGGCGCT 1392

** ** *** * ***** ***** ** * * ** ** * ***

ASDE TGAGACATTCCCTGAGATCGCTCTTCTCAACACCAAAGAACCAACGCATGTGAAGGGAGG 1766

4K GGG------CCCCTCGATCCTTTTCCTC--TTCCAACAAACC----CACTCCAA------ 1434

* *** **** * * *** **** **** ** **

ASDE GGTCCTAG-ACCTCACCTTTGCCACTGCAACAATGGTGGAGAGAATTCGGTGGTGTGTCG 1825

4K ----CTATTACCCCAAGTTTACC-CCGCA--------------GGCTGGGTGAAGCCCCG 1475

*** *** ** *** ** * *** * **** * **

ASDE ATGATACGGTTACTAGTGATCACTACGGCATAGTCACCACACTAATGGATGCAGGTCCAG 1885

4K GC--TCCTTCTTCT-----TCTCCA-GAAATTCTTTTCCTCCTCCTT-ATCCTTCTCCTA 1526

* * * ** ** * * * ** * * ** * ** * ***

ASDE CCCAAAGACCACACCACATTCCTAAATGGAAAACAGACAAGGCCAACTGGTTTGCCTTCC 1945

4K TCC-----CCACTC----TTCCC------------------CCCCTCTTCTTTACATTTT 1559

** **** * **** ** ** *** * **

ASDE AAGAAGGCTTGGCTCGATGTCTGAAAGACAATGAACCCAATAACAATAATGAAAACGTGG 2005

4K AGTCCATTTTAGTTCAGCCCACCCAAGGGAATCTCCTGTCCTGTGGTGCTGAAGG-GTCG 1618

* ** * ** *** *** * * **** ** *

ASDE ATGTGCTAGAGGCAAGGCTAATCC--AGGCCATAAACCAGGCAGCCTCACAAACCATTCC 2063

4K A-GTGTAGGAAACAGAG--AATTCTGAGGCCCTTCATAAGA---CCTCA------ACCCC 1666

* *** ** ** * *** * ***** * * ** ***** * **

ASDE CAAAACTCGTCCATGGTCCAGAACTCACAAAGACGCCTGGTACTATAATGACGAGATCAA 2123

4K TGAGAGTAGGA-ATAGGATAGACCTCCGAAAG------GGGACC-TAA---------CAA 1709

* * * * ** * *** *** **** ** ** *** ***

ASDE AGAGGTCAACCACCGGGTTAATATGTGTAGAAAAAACTTCCGACGGCAAAGATCTCCCGA 2183

4K AC---TTATCCTCTC--TCAATGACCACAAAATGAAC---------CAGA----CCATGG 1751

* * * ** * * *** * ** *** ** * * *

ASDE CAATCTGGCCCTGCTGAGGGAAGCTGTTGTGGATGCCAAGGAAACTACCAACAGAGTAAG 2243

4K CAGTC--ACCCTG-------AAGCCCCT--------CAAGG-------CTACAGGGGAGC 1787

** ** ***** **** * ***** * **** * *

ASDE GCAGGAAAAATGGCTGGAATG-GTGCCAGTCTTTTGGGTACCAGACCAGCCTTACAGAGT 2302

4K GAAAGAAA---GGC-AAAATGCACGCCGA----TTGTGT------CCGGCCT-GCAGAAA 1832

* * **** *** **** *** *** ** ** **** ****

ASDE TGTGGAAACGGGTCAGGCAAGCGACAAGCCGCCAA-GCCCCGAAATGCACTCATCATGAC 2361

4K TGAGCAA--GGACAAGACA---GAAAAATCATCAACATTCTG---TCCATTCA-CA---- 1879

** * ** ** ** ** ** ** * *** * * * ** *** **

ASDE CCACAATCAGAGGCAAATAGATTGGTGCTTGAGTTCTCTGCCAGAACCAGCACCAACAAT 2421

4K CCAGACTGAGAG--AAAT----------CTGAATTCCCC-TCAGAAT------------T 1914

*** * * **** **** *** *** * ***** *

ASDE CTGCCTCCAATGATGAGAGAAAAACAACAAAACTTAAATCCAGAAAGACTTGCT-CTCAT 2480

4K CT-----CAAAGTCAAGA-AGGGGC--TCAAGCTGAA--CCAGCCAAACCAGCTGCCCAC 1964

** *** * *** * * ** ** ** **** * ** *** * **

ASDE AAGAGACAAGGCACTCGAAGCTGATGAAGCAGATGCCTTGTTCTCTCTTAGGGAACTAAG 2540

4K ACTACTAAAGGCCTTAAACCCCTGCAAAGGGGAGGCGT-------------GAAGCGACG 2011

* * ***** * * * *** ** ** * * * * * *

ASDE AAAATCATACGAATCCAGTTCTGGGTCAGCACCGGGATCTGATGGGATCTCTCACCCCAT 2600

4K AAGGGTG---GAGTCTAAACTC---TTAGGA--GGAGTCTGAA-------CCCACCCC-- 2054

** ** ** * * ** * ** ***** * ******

ASDE CATTTCGCATCTAGGCCTTGCAGGAGAACTTGCATTCTTGCAGGTTATCTACAAATCCTG 2660

4K CAAATCGC--------------------CTAACGCTCTT-TAAGGTACCAAAAAGTCCTG 2093

** **** ** * **** * * ** * * ** *****

ASDE GCAAACAGCCACGGTGCCCCAGAGCTGGAAACAAGCCACAATAGTTCCCATCCCAAAGCC 2720

4K A-AGACTTCGACAATGCTTACCAGCTG--ATTAGGGCATTGGA----------------- 2133

* ** * ** *** ***** * * * ** *

ASDE AAAGGAGCCTGGCAAGTACCGCCCCATCTCTCTTCTCAGCTGCCTGGGTAAAACAGCTGA 2780

4K AAAGGAA--AGGCAAGT-CAGAC---TTTCAATCCGAATCTGT---------AGAGATGA 2178

****** ******* * * * * ** * * * *** * ** ***

ASDE GAAGATGGTACTCAACAGGCTCCGATGGAAAACGGGTCCCCCCCATGAACA-CCTGCACG 2839

4K GGGCATGATTATT-----GTCCCTAAAGATAAAG-----CTACCCTGAACTTCCTGCGGG 2228

* *** * * * ** * ** ** * * ** ***** ***** *

ASDE GGTTCACAAGGGGTAAGAGCACTGCTCATAGCATTTCCACACTCTTAAGCACAATCTGTA 2899

4K AAATCA-AGGTGCTAAAAG--------ATGG---------------GAGAAAAGTGAGTA 2264

*** * * * *** ** ** * ** * * * ***

ASDE CCTCGCCCGCCGTGGTTGTCTTCCTTGACCTGGAGAAGGCTTTTGAGTTGGCAAGTCCAC 2959

4K TCTCGCC-------ACTGACCTCC-----CAAGAGAAAAGAACCAAGATGGTG---CTAC 2309

****** ** * *** * ***** ** *** * **

ASDE TAGCCATTCAAGAGACCCTAATCCACAAAGGAGTCAAAGGCAGACTCTTGGCCTGGATAG 3019

4K TTGGCTTTC--------------CACTAGGG---------------TTTGATGTGGA--G 2338

* * * *** *** * ** *** **** *

ASDE CTGACTATTTTAAAAATAGATCAGCAAATGTCAGATTTCAAGGCCACCTCTCACAGCACA 3079

4K GTGATTACGTC-----TCTCCCA-CAAGTGGTGGA------GG---CTTCCCGCA----- 2378

*** ** * * ** *** ** ** ** * ** * **

ASDE TGCCACTTGAAAATGGAACTCCTCAGGGAGGGGTTCTTAGTCCAGCCCTGTTTAATACCC 3139

4K --CGACC---AAAGGGAAATCTTCAACCAGG----------CAAGTCCTGGT---CACCC 2420

* ** *** **** ** *** *** * ** **** * ****

ASDE TCATGTCCAACATACTCGACATTCACCTGCCAGAGGGATGCAAGATCATCTCTTATGCAG 3199

4K TCA------------------------------AGGGAGCCCCAATCACC---------- 2440

*** ***** * **** *

ASDE ATGACTTGGCAATCATAGCCTCTGGCAATCACTGCCTTACTAGAGCTCAGCGTTGTCTGA 3259

4K -------GGCAATCATAGCCTCTGGCAATCACTACCCAACTAGAGCTCAGTGCTGTCTGA 2493

************************** ** ************ * *******

ASDE ACCTGGTGTCCGAAGAGTGTTGTAGGACGGGTCTAAAAATATCAGCAGCAAAATCCAAAG 3319

4K ACCTGGTGTCTGAA--GTGTTGTAGGACGGGTCTAAAAATA---GCAGCAAAATCCAAAG 2548

********** *** ************************* ****************

ASDE CAATGGCTTTGAGAACCAATGTCAGAAACAAAAAACTCACTATACAGGGTATGGATCTGG 3379

4K CAATGGCTCTGAGATCCAATGTCAGAAACAAAAAACTCACTATACAGGGTATGGATCTGG 2608

******** ***** *********************************************

ASDE AATGAGTGAAGGATTATCTATACCTTGGTGTATGGATAGGACACACACTCACTTTCAAAA 3439

4K AATGGGTCAAGGACTACCTATACCTTGGTGTATGGATAGGACTCACACTCACTTTCAAAA 2668

**** ** ***** ** ************************* *****************

ASDE TGGAGATCCAATACCTGCTTGACAGAACCGAGGAAAGACCGTCAGTCATGAAAGTCTTGA 3499

4K AGGAGATCCAATACCTGCTTGACAGAACCAAGGAAAGACTGTCAGTCATGAAAGTCATGG 2728

**************************** ********* **************** **

ASDE CAGGGATACACATAGGAGCAGGACACAAAGTACTAAGATCATTCTATGTACATGCTGTCC 3559

4K CAGGGAGACACATAGGAGCAGGACACAAAGTACTAAGATCATTCTATGTACATGCTGTCC 2788

****** *****************************************************

ASDE GTCCTATTATTGACTATGCATCTGTCGCCCTCATTGCTTCCAGCACAACATTAAAAGAAA 3619

4K GTCCTATTATTGACTATGCATCTGTCGCCCTCATTGCTTCCAGCACAACATTAAAAGAAA 2848

************************************************************

ASDE AACTGGAAATAATACAAAACGAAGGAGCCAGAATAATTCTAGGTGCACCTAAGTGGACAA 3679

4K AACTGGAAACAATACAAAACGAAGCAGCCAGGATCATTCTAGGTGTACCTAAGTGTACAA 2908

********* ************** ****** ** ********** ********* ****

ASDE AGGTCATCAACCTCCTCATGGAAACTGATTTACCGTCCATGGACACTAGAATTGATCTAA 3739

4K AGGTCATCAACCTCCTCATGGAAGCTGATTTACCGTCCATGGACACCAGTATTGATCTGA 2968

*********************** ********************** ** ******** *

ASDE TGGTAGCACAATTCATTTCCAAGGTCCTGCAGGCACCCACAAACTCCTATCTAAGACAAA 3799

4K TGGTAGCACAGTTCCTTTCCAAGGTCCTGCAGGCACCCAGAAACTCTTATCTAAGACAAA 3028

********** *** ************************ ****** *************

ASDE GAGTTCTCAGACGCCTACAACAAGATTACCAGTTGTTTGCGGACAATTCCTGGCTCACAC 3859

4K GAGTACTCAGACGCCTACAACAAGTCTACCAGTTGTTTGCGGACAATTCTTGGCTCACAC 3088

**** ******************* *********************** **********

ASDE ATACAGCCAGAGTTTTAATACGCTTTCAGCTAAAACTTTCATTGCTTGCCAAGGGTATGG 3919

4K ATACAGCCAGAGTCTTGATGCGCTTTCAGCTAAAAGAATCTTTGATTGCCAAGGGCATGG 3148

************* ** ** *************** ** *** ********** ****

ASDE ACTCCCCTCACCCTGATTACAAAGAACCCC-ACCGTGAGCGGGGGTTCTTTGTAATCAGG 3978

4K GCTCCCCTCACCCAGACTACGAAGAACCCCCGCCGTGGG-------------TAAACGAA 3195

************ ** *** ********* ***** * *** *

ASDE GTGAGTCAAGGTAGTCGTTTTAGAATTCTCAGTTCTAAATCTCACAATGAGAAAAGATCA 4038

4K ATGA----------------TCGAATTCTCAATTCTAAATCTTACAATGAGAAAAGATCA 3239

*** * ********* ********** *****************

ASDE ATATTCCATGCCTATCCTAAAGGCACAAGCACAAAGGGTCATTGAACAAATAACTCCGCC 4098

4K ATATTCCATGCCTAGTCTAAAGGCACAAGTGCAAAGGGTCATTGATCAAATAACTCCTCC 3299

************** ************* ************** *********** **

ASDE GGGTAGTATAACTTACTACACGGACGGATCCGTGGATCCAATAAACCATACTGCAGGCGC 4158

4K GGGTAGCATAACATACTACACGGACGGATCTGTGGATCCCATAAACTATACTGCAAGCGC 3359

****** ***** ***************** ******** ****** ******** ****

ASDE CGGCTTTGCAACAAAGGATACCACAGCATCCATTAGGGTCACTGACAATGCCTCAACGCT 4218

4K CGGCTTTCCAACAAAGGATACCACAGCATCCATTAGGGGCACTGACAATGCCTCAACGCT 3419

******* ****************************** *********************

ASDE TCAGGCTGAAACGGTTGCGATCATGGAAGCATTGACACACGCGTCCCTGAGGGGAGGGCA 4278

4K TCAGGCTGAAACGGTTGCGATCATGGAAGCCCTGACACACGCGTCCCTAAGGGCAGGACA 3479

****************************** **************** **** *** **

ASDE CGTTGTCATTCATACAGATTCAAGAGCAACTATTGACAGTTTACAGCATAGCATGCCCCC 4338

4K CGTTGTCATTCACACAGATTCAAGAGCAACTATTGACAGCTTACAGCATAGCATGCCCTT 3539

************ ************************** ******************

ASDE AGATAACATCTACCTCTTGACAACAGTACTATACATAGCCCAAAGAATCCTTAGTCAAGG 4398

4K TGACAACATCTACCTCTTGACAACAGTACTAACCATAGCCCAAAGAATCCTCAGTCAAGG 3599

** *************************** ****************** ********

ASDE TAGAAGAATCATCATAAACTGGGTCCCAAGCCACATAGGCATACAAGGGAACGAGCTTGC 4458

4K TAGAAGAATTATCATTAACTGGGTCCCAAGTCACATTGGCATACACGGGAACGAGCTTGC 3659

********* ***** ************** ***** ******** **************

ASDE TGATAAACTAGCCGAAAAGGGCAGGGGTATGACCCCATCCTCCATGATCGTTAAACCCAG 4518

4K TGACAAACTAGCTGAAAAGGGCAGGGGTATGCCCCCATTCTCCATGATCGTTAAACCCAG 3719

*** ******** ****************** ****** *********************

ASDE CCGAAGGGGACTGAGCCAAGGTACCAAAGCTGCAGCGTGTGCGGTCCTCCGGGGAGCACA 4578

4K CCGAAGGGGATTAAGCCAAAGAACAAAAGCTACAGCGTGTGCGGTCCTCCGGGGAGCACA 3779

********** * ****** * ** ****** ****************************

ASDE TAGAGAAAACATCACAAAATCGCTCGCTGCCAAGTGGTACTCAGATGCTTCAGGCTATGA 4638

4K TAGAGAAAATATCACAAACTCGCTCGCTGCTAAGTGGTACTCAGATGCTTCAGGCTATGA 3839

********* ******** *********** *****************************

ASDE GCCACTGGCCCTTCCTCCAAATACAAAAAGAGGTACCGAAGTCATACTATTCAGACTAAG 4698

4K GCCACTGGCCCTTCCTCCGAATACAAAAAGAGGTACCGAAGTCATACTATTCAGTCTAAG 3899

****************** *********************************** *****

ASDE ACTAGGTTACCAATGCGCTTGGCAAATTATTGACAGTGAGTCTGGGAGGTCATGTAAACA 4758

4K ACTAGGTTACCAATGCA------------------------------------------- 3916

****************

ASDE CTGCGGCGAGCCTAACGCCACCCTGTTACATTACTTGCAGAATTGTGTACACACACAATT 4818

4K ------------------------------------------------------------

ASDE TCTGAGACAGGGACCACCCACCACAGCCGCCGGGCTGGTAAAAAGGGTGGGCAACATGCT 4878

4K ---------------------------------------AAGAGGGGTCTGTAACATGCT 3937

** * **** * ********

ASDE TTCACCATGGCAGCTGGATCGCTTGCTTGCAATCCAGCCACCGCGGTAAGCATGCAGTTC 4938

4K TACTACGTGGCAGCTGGATCGCTTGCTTGCAGTCCAGCCACCGCGGTAAGCATGCAGTTC 3997

* * * ************************ ****************************

ASDE AAAGAAAACATTTGAGTTAACTAGAAATATTTAACACAGGCCGGGCCACCCCAGAGAAGA 4998

4K AAAGAAACCATCTTAGTTAACTAGAAGTAGTTAACACAGGCCGGGCCACTCCAGAGAAAA 4057

******* *** * ************ ** ******************* ******** *

ASDE GGCCCGGGCGAAGCATGACTTCGCAAATCTAACT 5032

4K GGCCCGGGCGAAGCATGACTTCGCAAATCTAACT 4091

**********************************
